# Prison health situation and health rights of young people incarcerated in sub-Saharan African prisons and detention centres: a scoping review of extant literature

**DOI:** 10.1186/s12914-019-0200-z

**Published:** 2019-05-22

**Authors:** Marie-Claire Van Hout, Rosemary Mhlanga-Gunda

**Affiliations:** 1 Public Health Institute, Exchange Station, Liverpool John Moore’s University, Liverpool, L32ET UK; 2 College of Health Sciences, Centre for Evaluation of Public Health Interventions, Department of Community Medicine, University of Zimbabwe, Harare, Zimbabwe

**Keywords:** Sub Saharan Africa, Human rights, Prisons, Children, Juveniles, Adolescents, Availability and accessibility of health services, Availability of basic necessities, Human immunodeficiency virus infection (HIV)

## Abstract

**Background:**

Treatment and special protection of the rights of incarcerated young people in prisons are mandated under the Sustainable Development Goals (SDG), as well as under United Nations (UN) human rights instruments.

**Methods:**

A scoping review mapped what is currently known about prison conditions and health situation of detained and incarcerated young people in sub- Saharan African (SSA) prisons. A systematic search collected and reviewed all available and relevant published and grey literature. Following application of exclusion measures, 54 records remained, which represented 37 of the 49 SSA countries. These records were charted and thematically analysed.

**Results:**

The ages of children and adolescents held in SSA prisons ranged from 12 to 18 years. Three main themes were generated during the charting exercise; the prison environment for young people; availability and accessibility of basic necessities and navigating the prison system for health care and outside continuum of care.

**Conclusions:**

The review highlights the grave and continuing deplorable situation of young people held in SSA prisons. The violation of international human rights norms is observed in the systemic abuse and detention of young people with adults. Basic needs are not met in relation to sanitation, ventilation, safe spaces, protection from physical and sexual violence, clothing, food and access to HIV and medical care.

**Electronic supplementary material:**

The online version of this article (10.1186/s12914-019-0200-z) contains supplementary material, which is available to authorized users.

## Background

The global prison population continues to rise, with an increase in almost 20% observed between 2000 and 2015, despite the reduction in global crime trends [1]. Prison overcrowding, human rights abuses and growing numbers of vulnerable prisoner groups represent contemporary challenges for prison administration, and are underpinned by disproportionate use of pre-trial detention and imprisonment for non-violent or minor offences [1, 2]. The 2017 Global Prison Trends report [1] observed over 714,000 women and girls in prisons, and that the number of women in prisons globally had risen by 50% since 2000. This represents a significant rise in comparison to male prison populations which rose 20% in the same timeframe. Within this one third are on remand, and almost 20% of those convicted are in prison for drug-related crimes [2].

Available global data with regard to children in detention has estimated this cohort to be about 1 million in 2010, with an upcoming report by the UN Global Study on Children Deprived of Liberty intended in 2019. Most recently, Penal Reform International reported on regressive moves where some countries are reducing or reduced the minimum age of criminal responsibility in 2016, despite the unequivocal recommendation of the United Nations (UN) Committee on the Rights of the Child that this cut off should be no lower than 12 years, and the recommendation in 2016 that it be raised progressively to 18. For young people who are in conflict with the law, imprisonment should only be “a measure of last resort and for the shortest appropriate period of time” [3]. Key vulnerable populations of detained or incarcerated young people include; incarcerated girls; lesbian, gay, bisexual, or transgender (LGBT) youth; commercially sexually exploited youth; and ‘cross over’ youth involved in both the juvenile justice and child welfare systems [4]. The rate of conviction among girls has been greater than among adult women. In late 2015, the Special Representative of the UN Secretary-General on Violence against Children published the first report of its kind outlining the unique vulnerabilities of girls in the criminal justice system including histories of violence and abuse, poverty, unstable family environments, discrimination and presence of physical and psychological health conditions [2]. The report also suggested that some countries in effect use criminal justice systems as a substitute for weak or non-existent child protection systems [2].

Treatment and special protection of the rights of incarcerated young people in prisons are advocated for under the Sustainable Development Goals (SDG) (3,4,5,6, 8,10 and 16) which stress we will “leave no one behind”. They are also mandated under United Nations instruments presented in Table 1 [5–13].

**Table 1:** Treatment and special protection of the rights of incarcerated young people in prisons

Standard Minimum Rules for the Treatment of Prisoners (‘Mandela Rules’) (A/RES/70/175) (2016)[5]	
Standard Rules for Non-Custodial Measures (‘Tokyo Rules, 1990’)[6]	
Rules for the Treatment of Women Prisoners and Non-Custodial Measures for Women Offenders (‘Bangkok Rules, 2016’) (A/RES/65/229) [7]	
UN Convention on the Rights of the Child (1989) [8]	
UN Standard Minimum Rules for the Administration of Juvenile Justice (‘Beijing Rules’, 1985) [9]	
UN Guidelines for the Prevention of Juvenile Delinquency (‘Riyadh Guidelines’, 1990) [10]	
UN Guidelines for Action on Children in the Criminal Justice System (‘Vienna Guidelines’, 1997) [11]	
UN Rules for the Protection of Juveniles Deprived of their Liberty (‘Havana Rules’, 1990) [12]	
UN Guidelines for the Appropriate Use and Conditions of Alternative Care for Children (2009) [13].	

Despite the SDGs and these international guidelines, conditions pertaining to the ill treatment of young people in criminal justice systems across the world continue to warrant attention, with systemic abuse of detained young people, detention of young people with adults, and deplorable conditions continuing to be observed [1, 2].

In terms of health, young people in detention have unique and unmet medical needs (dental, reproductive, mental health, infectious illnesses), and may be disproportionately affected by learning disabilities, poorer mental health, risky health behaviours, self-harm, victimisation and suicide [4, 14–16]. They are medically vulnerable and face a disproportionately high morbidity and mortality rate compared to the general population [4, 14]. Being placed in prison environments and other closed settings exacerbates their existing mental health problems, learning difficulties and behavioural conditions. Incarceration exposes them to infectious diseases, trauma, violence and injury [4, 16], impairs positive child and adolescent development, and impairs transition to adulthood, and hinders successful re-integration into the community on discharge [6, 7, 15].

In the sub-Saharan African (SSA) region, basic rights for incarcerated or detained young people, as enshrined in the UN Convention on the Rights of the Child, are mandated in (amongst others) the African Charter on Human and Peoples’ Rights (1981) [17] and the African Charter on the Rights and Welfare of the Child (1999) [18]. Other statutes and conventions addressing rights of young people incarcerated and detained in Africa include: the African Youth Charter (2006) [19]; Declaration and Plan of Action for an Africa Fit for Children (2001) [20]; Kampala Declaration on Prison Conditions in Africa (1996) [21]; Principles and Guidelines on the Right to a Fair Trial and Legal Assistance in Africa (1999) [22]; Lilongwe Declaration on Accessing Legal Aid in the Criminal Justice System in Africa (2004) [23]; Lilongwe Commitment on Justice for Children (2009) [24] and OAU Convention Governing the Specific aspects of Refugee Problems in Africa (1969) [25]. Available data is limited regarding numbers of detained and incarcerated young people, who in 2008 were estimated to be approximately 0.5–5% of the total SSA prison population [26]. Of concern is that HIV prevalence in SSA prisons has been estimated at two to 50 times that of non-prison populations [27, 28]. In SSA prisons, a 2016 estimate reported that over 668,000 people are incarcerated, with women and girls overall having a higher prevalence of HIV than their male counterparts [29]. Adolescent girls in SSA are identified by the World Health Organization (WHO) in 2016 as a key population particularly vulnerable to HIV infection [30]. In 2016, new infections among girls and young women aged (15–24) were 44% higher than their male counterparts.

Research activity on SSA prison populations, HIV prevalence and their health situation remains fragmented in the region [31]. Telisinghe et al. [29] in their 2016 Lancet article underscore that most countries in the SSA region do not collect strategic information on incidence, prevalence, or clinical outcomes of HIV and TB infection in prisoners, despite the African continental epidemic spanning a host of key populations at risk of HIV acquisition. We build here on a larger scoping exercise undertaken [31] within the support of a Medical Research Council (MRC) grant investigating prison health in the SSA region, and present a unique and extensive mapping exercise of extant information on juvenile prison conditions, health needs and rights in SSA prison settings.

## Methods

Scoping reviews are a research synthesis which maps literature on a particular topic or research area, and provides an opportunity to identify key concepts; gaps in the research; and types and sources of evidence to inform practice, policymaking, and research [32]. For insufficiently researched topics such as this, scoping reviews are particularly useful as they include a wide range of data across identified sources and designs, and are used to raise awareness, and inform policy and practice [32–34].

The review process commenced with the establishment of the joint author team, who have relevant expertise in public health, prison health, and community medicine in Africa. We adhered to a previous similar scoping methodology [please see 31]. The underpinning research question was; ‘What is known in the literature about the prison conditions, health situation and unique health rights of young people in contemporary sub-Saharan African prisons?’ The term “prison” was adopted as representing facilities housing both on-remand young people and convicted juvenile prisoners. These settings included regular prisons, police holding cells, pre-trial detention, closed youth institutions, and camps where people who use drugs are forced into mandatory labour as means of rehabilitation. We restricted the scoping exercise to all records reporting on the situation for young people detained when in conflict with the law and under the age of 18 years [15]. We excluded literature on infants and babies incarcerated with their mothers, which are presented in a specific scoping review elsewhere not yet published.

The six-stage iterative process [34] was closely followed by the team, and consisted of (1) identifying the research question, (2) identifying relevant studies, (3) study selection, (4) charting the data, (5) collating, summarizing and reporting the results, and (6) an international expert advisory review exercise. Search terms were generated, and combined with SSA region. The general search strategy is illustrated in Table 2.

**Table 2:** Search Terms and Strategy

Key Word	Alternative
Juveniles in Prisons	Juveniles in prisons*, OR Juvenile inmates *, OR juvenile prisoners *, OR incarcerated juveniles *, OR Children in Conflict with the Law *, Adolescents in prisons*
Research evidence	AND physical environment*OR availability of basic necessities*OR availability of adequate and quality nutrition* OR availability and accessibility of healthcare*OR availability of health education and promotion services and sexual reproductive health* OR availability of HIV/AIDS prevention* OR availability and accessibility of counselling services * OR availability of psychosocial services *
African Countries	Sub Saharan Africa*OR Africa*OR and the names of all the individual countries in Sub Saharan Africa
1 Juveniles in prisons	
2. Juvenile inmates OR Juvenile inmates OR Juvenile prisoners OR incarcerated Juveniles OR children in conflict with the law OR Adolescents in prisons	
3. OR physical environment, OR availability of basic necessities OR availability of adequate and quality nutrition, OR health services availability and accessibility, OR availability and accessibility of health care, OR availability of health education and promotion services and sexual and reproductive health, OR availability of HIV/IDS prevention, OR availability and accessibility of counselling services, OR availability and accessibility of psychosocial services) AND	
4. Africa Databases were searched using the appropriate subject headings and/or keywords or text words for the above search groups:	
Sample Search (Pubmed Central) searched on 15-10-2018 # Searches Results	
1. Juvenile inmates OR Juvenile inmates OR Juvenile prisoners OR incarcerated Juveniles OR children in conflict with the law OR Adolescents in prisons	
2. OR physical environment, OR availability of basic necessities OR availability of adequate and quality nutrition, OR health services availability and accessibility, OR availability and accessibility of health care, OR availability of health education and promotion services and sexual and reproductive health, OR availability of HIV/IDS prevention, OR availability and accessibility of counselling services, OR availability and accessibility of psychosocial services) AND Africa 1504	

The search was conducted by author two between October and December 2018 using university databases at the University of Zimbabwe and Liverpool John Moore’s University, PubMed Clinical Queries, and Scopus (exploratory search with selected references downloaded for the purpose of clarifying search terms), and with support from a university librarian. Comprehensive searches were subsequently conducted in the Cochrane Library, PubMed, Science Direct, EMBASE, EBSCO, Medline, PsycINFO and CINAHL, and restricted to the time period 2000 to 2017. No limitations on language were applied.

In order to ensure full coverage of current knowledge and perspectives relating to juvenile health situation in SSA prisons, we included international and national policy briefs, documents and reports, country situational assessment reports, conference proceedings, news reports, commentary pieces and editorials, in addition to empirical peer-reviewed scholarly literature. Records included had either young people detention or prison centres in SSA or the papers would report on adults incarceration conditions but with a young offenders section included. Where possible we included studies, which observed or described prison staff experiences and perspectives on young people incarcerated in SSA prisons. Follow-up search strategies included hand searching of reference listings. Hand searches were conducted on international aid and development organisational websites, health, medical and human rights related databases, and websites of country governments and non-governmental bodies.

**Fig. 1:**
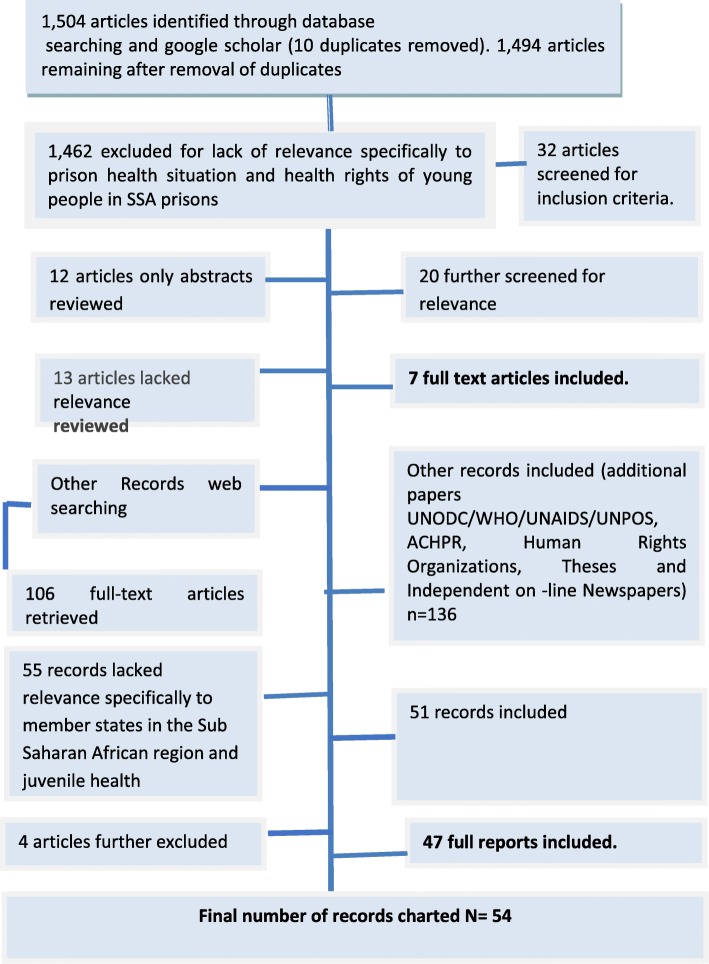
Flowchart for inclusion and exclusion of literature

All records were managed using EndNote. Screening was undertaken by author two, and cross checked by author one. The title and abstract of each record were initially screened by the author two, with both authors independently reviewing included and excluded records to determine inclusion status. All records warranting inclusion by the team were then procured for full text review. Where required records were translated into English. A second screen of the full-text of each record was conducted by the team. Studies were excluded at this stage if found not to meet the eligibility criteria. Figure 1 reflects inclusion and exclusion criteria used to chart the studies.

Following application of exclusion measures, 54 records were charted and thematically analysed, as per scoping review protocols. This involved the creation of a spreadsheet used to chart relevant data (data collection categories were the year of publication, author, location, method and aim, key findings and conclusion to enable the identification of commonalities, themes, and gaps in the literature). Charting involved collecting and sorting key pieces of information from each record. The team conducted a trial charting exercise of five records as recommended by [33], followed by a joint consultation to ensure alignment with the scoping question and its purpose. The charting exercise generated specific themes pertaining to juvenile health situation and health rights in prisons in the SSA region. Disagreements around theme allocation were resolved through team discussion.

## Results

The scoping exercise revealed a limited evidence base within SSA pertaining to incarcerated or detained young people and health situation. Most included records originated from human rights organizations and annual reports from United States Department of State Bureau of Democracy, Human Rights and Labor and the African Commission on Human and Peoples’ Rights. There is a dearth of empirical peer-reviewed scholarly literature. The ages of young people incarcerated or detained in the region ranged from 12 to under 18 years. Evidence was found in 37 of the 49 SSA countries highlighted in Tables 3 and 4, and with 11 of those referring to juvenile detention centres.

**Table 3:** Sub-Saharan African (SSA) countries

Angola	**Côte d'Ivoire**	**Madagascar**	**Seychelles**
**Benin**	Djibouti	**Malawi**	**Sierra Leone**
**Botswana**	**Equatorial Guinea**	**Mali**	
Burkina Faso	**Eritrea**	**Mauritania**	**Somalia**
**Burundi**	**Ethiopia**	Mauritius	**South Africa**
**Cameroon**	**Gabon**	**Mozambique**	Sudan
**Cape Verde**	The Gambia	**Namibia**	**Swaziland**
**Central African Republic**	**Ghana**	Niger	**Tanzania**
**Chad**	Guinea	**Nigeria**	**Togo**
**Comoros**	**Guinea-Bissau**	Réunion	**Uganda**
**Congo (Brazzaville)**	Kenya	**Rwanda**	Western Sahara
Congo (DemocraticRepublic)	**Lesotho**	**Sao Tome and Principe**	**Zambia**
	**Liberia**	**Senegal**	Zimbabwe

Countries in **bold** present records included in the review

**Table 4:** Summary table of records per country

Country	Number of Records chartered
Zambia	6
Mozambique	2
South Africa	2
Lesotho	1
Nigeria	3
Côte d’Ivoire	2
Somalia	1
Ghana	2
Eritrea	1
Benin	1
Malawi	2
Burundi	1
Liberia	1
Chad	1
Namibia	1
Uganda	1
Cameroon	1
Cabo Verde	1
Ethiopia	1
Central African Republic	1
Mali	1
Guinea Bissau	1
Comoros	1
Republic of the Congo	1
Gabon	1
Mauritania	1
Sao Tome and Principe	1
Senegal	1
Sierra Leone	2
Tanzania	1
Togo	2
Madagascar	1
Equatorial Guinea	1
Rwanda	1
Swaziland	1
Seychelles	1
Botswana	1
Total	51*

Summaries and characterisation of the chartered results are found in the Additional file 1: Table S1. Table 4 presents a summary of number of records per country. * Three charted results not included in Table 4 are located in Sarkin in 2008 [38], the African Union 52nd session meeting in Côte d’Ivoire in 2012 [39] and Telisinghe et al. in 2016 [29] where the authors give a commentary on the literature review results on the status of penal institutions in Africa as a whole with some reference to certain countries within Sub-Saharan Africa.

Three main themes were generated during the charting exercise, namely the prison environment for young people; availability and accessibility of basic necessities, and navigating the prison system for health care and outside continuum of care. Where possible in this paper, we present illustrative narratives from the three qualitative studies with extended quotes [35–37].

### Theme one: the prison environment for young people

#### Overcrowding, unhygienic conditions and poor sanitation

Most penal institutions in SSA were reportedly built in the pre-colonial era and are still failing to meet even the most basic minimum standards for adults, with young people equally disadvantaged, and a significant shortfall in meeting international standards for juvenile detention. [38–42]. Incarceration conditions within countries were reported by the 2017 United States Department of State Bureau of Democracy, Human Rights and Labor to vary significantly [43–45]. Annual reports in 16 SSA countries (Central African Republic, Mali, Guinea Bissau, Comoros, Democratic Republic of Congo, Côte d’Ivoire, Equatorial Guinea, Gabon, Madagascar, Mauritania, Rwanda, Sao Tome and Principe, Senegal, Sierra Leon; Tanzania, Togo and Cape Verde) by the Department of State from 2012 to 2017 reported on harsh penal conditions described as potentially life threatening for young people [40–42, 44–57]. Official missions by the Special Rapporteur on Prisons, Conditions of Detention in Africa (referred to hereinafter as the Special Rapporteur) of the African Commission on Human and Peoples’ Rights (ACHPR) in Namibia, Uganda, Mozambique, Malawi, Cameroon and Ethiopia in the years 2001, 2002 and 2004 reported on poor penal conditions for detained and incarcerated young people, and underscored concern that young people endure the same inhuman and overcrowding conditions as their adult counterpart prisoners [58–63].

Eleven countries reported on juvenile detention centre conditions from 2001 to 2017 (Cape Verde, Lesotho, South Africa, Eritrea, Ghana, Mauritania, Nigeria, Swaziland, Togo, Rwanda and Zambia) [37, 44, 45, 56, 57, 64–73]. In 2004, some juvenile detention centres in South Africa were not overcrowded, but this was not uniform across all prisons holding young people as reported by the Special Rapporteur on Prisons, Conditions of Detention in Africa [64]. The Special Rapporteur on its mission to South Africa in 2004 reported that young people were held three at a time in single cells designed to accommodate only one person (in single cells measuring 3 m × 7 m) [64]. It was reported in 2004 by another investigation that young males at a Gauteng Correctional Centre were staying in communal cells and with no overcrowding observed [71]. In 2006, Liberian young people were reported to be held in tiny overcrowded cellblocks with between two and five other youth, and it was impossible for multiple prisoners to sleep lying down at once [65]. In Togo in 2014 overcrowding was reported in tiny cellblocks, which rarely exceeded 6 m × 5 m [56, 74].

A Zambian study by Topp et al. in 2016 [65] reported on similar overcrowded conditions in a youth detention centre. The process of transfer of young people to the facility was observed to be lengthy and protracted. In 2013 in Nigeria, Atilola reported overcrowding at the youth detention centres (known as ‘borstal homes’ [66]. This was also observed by Stout in 2001 in Lesotho youth detention centres [67]. In comparison to boys, in 2001 girls in Burundi were housed in adult women’s wings, an arrangement that reportedly helped ensure a degree of protection for them [75]. In 2011, girls in Ghana were held in the Girl’s Remand Home that was located on the same compound as that of the boys [68].

In 14 SSA countries (Zambia, Sierra Leone, Liberia, Togo, Burundi, Ghana, Lesotho, Côte d’Ivoire, Chad, Nigeria, Malawi, Somalia, Benin and Mozambique), evidence from studies, human rights organizations and investigative journalism reports in the timeframe 2000 to 2017observe that young people in conflict with the law are detained and incarcerated in dilapidated, substandard and inhumane physical environments, with poor ventilation, inadequate or non-existent lighting and severe overcrowding [29, 35–37, 66–68, 74, 76–84]. In 2012, commenting on the state of prison infrastructure described as old and dilapidated, a Mozambican boy said “… *As paredes estâo cansadas’* [The walls are tired]…” [84].

Detention of young people in prisons and detention centres beyond maximum capacity results in severe overcrowding, a known public health factor conducive to the spread of infectious conditions via risk environments and risk behaviours. This is duly acknowledged in a 2011 Zambian study:

“ … Prison confinement can increase vulnerability to HIV due to frequent unprotected sex in the form of rape, non-availability and non-use of condoms, as well as high prevalence of STIs … ” [85].

In Zambia in 2010, young people incarcerated at one of the prisons were reported to be sharing living quarters with those in the TB isolation cell [35]. Commenting on the fear of contracting TB, a 17-year-old boy said;

“ … I am worried I will catch TB. There is no window, just a small opening with wire over it—not much ventilation, there were … 23 TB patients in my living area. There are no vents, no air. I’m worried … .” [35].

Non-observance and non-implementation of infection prevention and control measures were also reported in Zambia in both 2011 and 2016 [29, 85], in the Central African Republic in 2012 and Equatorial Guinea in 2017 [41, 46] where isolation of patients with infectious diseases such as typhoid and TB was not practiced.

Poor sanitation and hygiene were consistently reported across all records. Conditions were characterized by insufficient, overflowing, non-functional toilets and bathing facilities with some water points close to sanitation outflows, and bathing buckets sometimes used as toilet facilities in the night. This was reported from 2001 to 2017 in Zambia, Sierra Leone, Mauritania, Central African Republic, Mali, Guinea-Bissau, Comoros, Democratic Republic of Congo, Gabon, Sao Tome and Principe, Senegal, Tanzania, Madagascar, Swaziland, Cape Verde, Malawi, Mozambique, Uganda, Cameroon, Ethiopia, Burundi, Lesotho, Côte d’Ivoire, Chad, Benin, Togo, Somalia and Eritrea [35, 39–41, 43, 46–53, 56, 57, 59–62, 65, 66, 68, 69, 71, 75, 79–81]. Such poor sanitation and hygiene was reported to exacerbate the spread and prevalence of body lice, scabies or other skin infections, respiratory complaints, diseases, diarrhoea and other preventable diseases [37, 46, 69, 77]. In Togo for example, it was reported that the Togo Brigadier Facility for minors had poor sanitation facilities and lacked portable water [56].

Lack of cleaning detergents and soap were reported to compound unsanitary and unhygienic conditions in Zambia, Sierra Leone, Central African Republic, Mali, Guinea-Bissau, Comoros**,** Gabon, Madagascar, Côte d’Ivoire, Swaziland, Malawi, Mozambique, Uganda, Cameroon, Ethiopia, Somalia, Togo and Chad [35, 40–42, 47, 48, 50, 51, 55, 56, 58, 60–63, 70, 74, 77, 82, 85] across the years 2001 to 2017. Lack of and/or erratic supplies of potable water affected prisoners’ hygiene in prisons in Zambia, Sierra Leone, Central African Republic, Mali, Guinea Bissau, Comoros, Madagascar, Senegal, Côte d’Ivoire, Swaziland, Mozambique, Uganda, Cameroon, Chad, Togo, Somalia, Malawi, Ethiopia and Eritrea [35, 40, 41, 47, 48, 51, 53, 55, 56, 58, 60–63, 70, 74, 77–79, 81, 82, 85] was reported in the same time period. Basic items like soap, and detergents for washing clothes were reported to be provided to incarcerated children in Cape Verde and Durban, South Africa [57, 64].

#### Mixing of young people and adults in same prisons

Holding conditions of incarcerated or detained young people varied within and among SSA countries. These ranged from the separation of young people from the adult population, to partial or no separation at all. Based on the annual human rights reports by the Department of State, 17 countries (Sierra Leone, Mauritania, Rwanda, Central African Republic, Mali, Guinea-Bissau, Comoros, Democratic Republic of Congo, Gabon, Sao Tome and Principe, Senegal, Tanzania, Madagascar, Swaziland, Côte d’Ivoire, Seychelles and Cape Verde) [40–42, 44, 45, 47–55, 70, 87] across the years 2012 to 2017 were observed to incarcerate young people with the adult population in their penal institutions. The mixing of young people with adults was reported in Burundi, Ghana, Zambia, Sierra Leone, Somalia, Chad, Nigeria and Côte d’Ivoire [29, 36, 68, 75, 77–80, 82] across the years 2002 to 2015. In 2017, young people in Tanzania and Botswana were mixed with adults during the day and while being transported to court [43, 54]. In the same year, young people in Equatorial Guinea were observed to have separate sleeping quarters and bathrooms to adult inmates, but with a shared common area for meals [46]. Some disturbing practices were observed in Ethiopia, Democratic Republic of Congo and Senegal in the years 2004 and 2017 respectively where some prisons facilitated easy access to juvenile quarters by adults, unlocked entryways and poor supervision by prison staff [49, 53, 63]. Young people were not housed with the adult population in only two SSA countries, South Africa, and Lesotho [64, 67, 71].

The practice of mixing young people with the adult population in penal institutions in most SSA countries was attributed to lack of resources to house minors separately [38]. In the years 2001, 2002 and 2004, this lack of prison resources to cater for minors was emphasised by the Rapporteur on Prisons, Conditions of Detention in Africa in Namibia, Mozambique, Malawi, Cameroon and Ethiopia [58, 59, 61–63]. In 2010, an officer in charge of a Zambian prison gave his opinion:

… “As a father it pains me that children do not have their own facilities … —we need to build a separate area for juvenile offenders … ” [35].

In 2011, a different Zambian study reported on the intimidation of young people, if they revealed the combined sleeping arrangements to formal investigators. A boy said:

“ … We sleep with the adults, but they told us to say we sleep in a juvenile cell. If we don’t say we sleep in a separate cell, they will beat us. We are given punishment when we start talking. But we are scared we might die here … ” [36]

#### Sexual abuse

The continuous threat of physical and sexual violence against young people was reported to be prevalent in SSA prisons. In 2016, Topp et al. [65] reported on the vulnerability of youth in SSA prisons due to lack of personal or family support, meaning they have a lack of food and other basic necessities, leaving them vulnerable to manipulation by wealthier and more powerful adult inmates who may prey on them sexually. From 2001 to 2012, physical and sexual abuse perpetrated by police, prison officers and adult prisoners on detained young people is evident in reports from Zambia, Mozambique, Uganda, Burundi, Côte d’Ivoire, Nigeria, Malawi and South Africa [29, 36, 58, 60, 61, 71, 75, 79–81, 85]. In 2017, in Swaziland in spite of young people being accommodated at youth correctional facilities, there were reports of inhuman and degrading treatment which included physical assault and strip searches of female young prisoners [70]. The mixing of young people with adult prisoners was observed to heighten exposure of young people to extreme physical and sexual abuse [38, 39]. Across the years 2001 to 2011, this abuse was observed to be present in police detention at the hands of the police or other detainees, during remand and after conviction by prison officers, adult prisoners or other young people [36, 37, 58, 64, 71, 85]. In 2004, South African staff at a juvenile correctional facility reported the prevalence of “male rape**”** in the juvenile section, with a frequency of about two to three reports a week [64]. In 2001, the Rapporteur on Prisons, Conditions of Detention in Africa in Uganda documented complaints that young people were victims of sexual assaults by other prisoners, but that prison authorities were ignoring the victims’ reports, with similar reports were made in Zambia and Malawi [36, 58, 60].

In 2001, it was observed that young prisoners in Namibia would agree to pair with adult prisoners in the secret hope that they would see their living conditions improve [59]. A Zambian detainee in 2010 described how adults would seek to establish relationships with young people, with failure by prison authorities to protect them;

“ … Mainly the juveniles are very vulnerable. As young people coming into prison, we are full of fear. The convicts take advantage of us by providing us with food and security. We enter their dragnet, but by the time we discover this it is too late … ” [35].

Across the years 2004 to 2017 adults in Benin, Ethiopia, the Democratic Republic of Congo and Senegal were observed in juvenile quarters with permission granted by the head of the prison [49, 53, 63, 86]. In 2001, a disturbing observation was reported by young people to the same Special Rapporteur in Zomba, Malawi [58]. They complained that prison officers themselves were engaged in trafficking them in exchange for money through transfers to the adult units where they would be abused by adult prisoners. The Special Rapporteur recommended that Malawi authorities should take up the issues of sexual abuse raised by the young people and in particular ensure that separation of adults and young people was strictly enforced, with punishment to all prison officers guilty of transferring young people into adult sections or the trafficking of young people [58]. Commenting on these sexual activities, a boy in Zambia shared his experience:

“ … Forced sexual activity is very common. The way we sleep, we are in one another’s lap. ” [36].

This is concerning given the risk of HIV infection. Data on HIV infection in SSA countries among detained young people is limited, and often dated. In 2001, a Zambian study reported an overall HIV prevalence of 27% among prisoners, with those under 20 years of age having a prevalence rate of 14.5% [88]. A 2017 Zambian study by Kumwenda et al. [37] reported that the prevalence of sexually transmitted infections (STI) among young people was attributed to sexual violence by adults during remand and in prison.

### Theme two: availability and accessibility of basic necessities

#### Inadequate bedding, linen and mosquito nets

Lack of adequate bedding, linen and uniforms, with young people sleeping on bare floors in their own clothes or using cartons as bedding was reported in Zambia, Sierra Leone, Central African Republic, Democratic Republic of Congo, Côte d’Ivoire, Swaziland, Equatorial Guinea, South Africa, Liberia, Ghana, Lesotho, Nigeria, Eritrea, Ethiopia and Cape Verde [35–37, 41, 46, 49, 55, 64, 68–71, 77, 79–81, 84] across the years 2001 to 2017. In contrast, at a Durban youth detention centre in South Africa in 2004, the girls section had beds [64]. Detainees in Zambia in 2010 were observed to be sleeping up to five young people on a mattress, covered with dirty unwashed blankets and with mattresses full of lice and dust [35]. In 2001 young people in Lesotho prisons slept on torn mattresses [67] and a similar observation was made at youth correctional facilities. In malaria endemic countries such as Zambia and Sierra Leone, no mosquito nets were provided to young people, while in Côte d’Ivoire only a handful of torn mosquito nets were available but not adequate enough to go around [35, 78, 79].

In 2010, in Zambia it was reported that remanded prisoners were not provided with uniforms, while convicted prisoners’ uniforms for young people were reportedly grossly inadequate [35]. Similarly, young people in Liberia in 2006 reported that they had no change of uniforms and were still wearing the same clothes since admission many months ago [76] while in the Central African Republic in 2012, the International Committee of the Red Cross (ICRC) and other religious groups supplied clothes to the prisoners [41]. In 2001, young people in Lesotho described the blankets and jerseys that they were provided with as “dilapidated”, and complained of suffering from ailments such as coughing, fever and stomach ache which they attributed to inappropriate clothing and cold baths in the winter [67]. In 2017, a boy in a Zambian prison said;

“ … As for me when I came here, after three days, I was surprised to find that I had a lot of rashes over my neck and body. I think even exchanging bathing items, when your friends use it and then you also use it also causes rashes … ” [37].

#### Poor quantity and quality of food

Food provided to young people was generally reported to be nutritionally insufficient in terms of quantity and quality, and described as barely edible and monotonous [39]. Lack of sufficient food rations coupled with poor quality food was reported in Zambia, Malawi, Mozambique, Namibia, Cameroon, Liberia, Burundi, Lesotho, Chad, Nigeria and Eritrea [29, 35, 36, 58, 59, 61, 62, 67, 75–77, 80] across the years 2001 to 2013. Similarly, the Department of State in its annual reports from 2012 to 2017 reported insufficient and poor quality food in some SSA penal institutions housing minors in Sierra Leone, Mauritania, Central African Republic, Mali, Democratic Republic of Congo, Gabon, Sao Tome and Principe, Senegal, Tanzania, Swaziland, Equatorial Guinea and Togo, with reliance on philanthropic organizations or relatives of incarcerated young people to supplement food allocations [40–42, 44, 46, 49, 50, 52–54, 56, 70]. In 2010, young people in a Zambian prison study [35] described the health consequences of food insecurity, describing symptoms such as irritability, sleep disturbance, burning pain, muscle atrophy and muscle cramps that are consistent with thiamine deficiency (vitamin B1). In 2011, officers in Zambian prisons reported cases of malnutrition related illnesses and deaths due to inadequate food [85]. In contrast in Ghana, the Rapporteur on Prisons, Conditions of Detention in Africa mission in 2014 observed that food provided to young people was of better quality than that provided to the adult prisoners [72].

Sarkin [38] and ACHPR [39] have underscored that in the face of a shortage of resources such as food, young people resort to competing with the general adult prison population for survival. Records dating from 2001 to 2011 in Zambia, Namibia and Malawi reported that young people were engaging in sexual transactions for food and other basic necessities not provided by the prison [36, 58, 59, 85]. Todrys and Amon’s 2011 study [36] reported that a 17-year-old Zambian male described how adult inmates seek to establish relationships with young boys and how prison authorities were failing to protect them, with a lack of follow up by staff on duty common. In one case the cell captain intervened by removing the man from the cell. A Zambian boy in this study said;

“ … We have had experiences where the older inmates become physical and abuse us, even sexually … I haven’t physically been abused, because I know the system, and avoid enticements. But my more vulnerable friends fall prey. Once you eat the food, they reprimand you, say you have no choice. I have seen it happen … ” [36].

The ACHPR [59] Mission to Namibia in 2001 also noted prison guards regarded these instances with indifference. Similarly, in Malawi adult prisoners were reported to help young boys with food and a place to sleep, before abusing them and using them as their “wives” [56].

### Theme three: navigating the prison system for health care and outside continuum of care

#### Prison healthcare provision and access to prison health care

Standards of health care provision for young people as for adults were inadequate and described as alarmingly poor in some SSA countries [56, 78]. Under-funding of prisons by governments has impacted negatively on provision and access to health care in penal institutions [37–39, 42, 62, 73, 80, 85]. In fifteen SSA countries (Sierra Leone, Mauritania, Central African Republic, Mali, Comoros, Democratic Republic of Congo, Gabon, Sao Tome and Principe, Senegal, Madagascar, Côte d’Ivoire, Equatorial Guinea, Swaziland and Togo), the Department of State in its annual reports from 2012 to 2017 observed that health care facilities in prisons when available, were characterized by inadequate resources such as shortage of staff, essential medicines, medical equipment, and poor health education and promotion (HEP) services [40–42, 44, 46, 48, 50–53, 55, 70]. Annual reports from the Department of State from 2012 to 2017 indicate that conditions had not improved in the majority of countries. In Benin and Guinea-Bissau in 2004 and 2017 (respectively), prison-based health care was described as virtually non-existent [47, 86]. While the majority of prisons lacked primary health care facilities and provision of services for the treatment of minor ailments, these were available on site in Lesotho, South Africa, Côte d’Ivoire, Zambia, and Ghana in reports dating from 2001 to 2017 [29, 36, 55, 64, 67, 68, 73]. Whilst in 2016 South Africa had on-site clinics, it was observed that staff were not adequately trained in primary care or preventive medicine [29]. Similarly, medical care in Ghana in 2014 was being provided by prison aides and not medically trained professionals [72], while in Chad other prisoners provided care to their ill peers [77]. In Zambia and Mozambique (in 2010, 2011 and 2012) the shortage of essential medicines resulted in young people not being cared for according to standard recommended treatment protocols, but with whatever medicine was available at the time the young person presented at the prison health facility (for example, use of paracetamol in treating all conditions) [35, 37, 84, 85].

Young people were reported to face the same challenges in accessing of health care as their adult counterparts [38, 39]. In 14 SSA countries (Zambia, Malawi, Mozambique Namibia, Uganda, Cameroon, Ethiopia, Burundi, Benin, Chad, Somalia, Nigeria, Ghana and Togo) poor access for young people to on-site medical clinics in prisons were reported [29, 36, 37, 58–63, 73–75, 77, 81, 82, 85, 86] across the years 2001 to 2016. Dependence on prison officers not medically trained to give permission for accessibility to healthcare staff was observed. Young people incarcerated in Zambia in 2011 said;

“ … Sometimes it is difficult getting to the clinic, sometimes you may not get to go. We ask the cell leader – [and even if they agree] the guards might say no … ” [36].

“ … If you are sick, then you can’t go to the clinic … ” [36].

In 2017, the Zambian Nakambala Approved Correctional School did not have a screening facility, or health care service and all young people were referred to the nearby clinic irrespective of the severity of the presenting condition [37]. This was observed to compromise their privacy and confidentiality during consultations. In 2017 in Côte d’Ivoire prisoners had to rely on guards to allow them to see medical staff at night within the prisons [55]. In Nigeria, Bella et al. in their 2010 study [73] of young people in the Ibadan remand home reported on the lack of adequate health facilities, and the prescence of anxiety, suicidal and depressive symptoms among participants.

#### Accessibility to continuum of care outside prisons

Across all records, delays and barriers to accessing outside medical care were observed. Delays of up to several days in accessing higher levels of medical care for severely ill young people were reported in Zambia, Central African Republic, Mozambique, Cameroon, Côte d’Ivoire and Eritrea across the years 2001 to 2017 and attributed to administrative barriers, lack of transport, fuel and security fears [35, 41, 55, 61, 62, 69, 85]. In 2002 in Cameroon, mandatory payment to community-based health care centres negatively affected access to medical services for referred sick inmates, despite access to treatment being supposedly free [62]. The same observation was made in Côte d’Ivoire in 2017 where philanthropic organizations paid for the medical care of referred inmates [55]. In Zambia in 2011 negative attitudes of medically unqualified and untrained prison officers controlled and evaluated the necessity for referral for onward management [36, 85]. In 2011 a 17-year-old Zambian boy said

“ … I asked for help at the clinic and they said they would take me to the hospital – that was seven months ago. They gave me some medicine but it only makes me sleep, it doesn’t help me breathe … ” [36].

#### Health education and promotion, sexual and reproductive health, psycho-social and HIV counselling services

Despite the enhanced risk of STI, TB and HIV acquisition in prisons, there was no evidence from the majority of countries in SSA that they provided key psycho-social services underpinned by health education and promotion (HEP), sexual and reproductive health (SRH), psycho-social and HIV counselling services to detained or incarcerated young people. Zambian prison policy in 2011 was reported to acknowledge the fact that penal environments exacerbate vulnerability to HIV infection due to prevalence of frequent unprotected sex, rape, laws that prohibit condom availability and distribution, and the high prevalence of STI in the adult prison population. As young people are detained in the same overcrowded prisons and mixed with adults, their exposure to disease is heightened [85]. Commenting on the lack of youth-friendly services at a primary health clinic and how this affected service uptake and health seeking behaviour by adolescents, a key informant shared her experience in 2017 as follows;

“ … At the clinic where juveniles are referred, there are no adolescent health services. This is a big challenge as some adolescents are shy to openly talk about their sexual related challenges. Such fears worsen their health … ” [37].

Information, Education and Communication (IEC) materials on HIV/AIDS and HEP on SRH and counselling services were documented as provided to young people in prisons located in Namibia, Cameroon, Nigeria and South Africa [59, 62, 71, 80] in 2001, 2002, 2004 and 2013 respectively. In 2004 and 2011 (respectively) psychologists and social welfare officers were reported to be available to incarcerated young people in South Africa and Ghana [64, 66, 72]. Counselling services that included SRH and HIV prevention, treatment and care were reportedly available to detained or incarcerated young people in South Africa, Uganda, Ghana and Zambia [37, 60, 66, 72] in reports dating across 2001 to 2017. Quality was compromised by the lack of trained staff for information provision, lack of available evidence-based HEP materials, and dissemination strategies [37]. Despite HIV counselling services being available in Zambia, low uptake of HIV testing among young people was reported [85]. Psychological services were not available in Democratic Republic of Congo, Namibia, Somalia, Ghana and Nigeria [49, 59, 73, 80, 82] across the years 2001 to 2017. Within the majority of countries, observations on the availability and accessibility of mental health and psychiatric services for incarcerated young people were not made. In 2014 the Office of the High Commissioner for Human Rights United Nations Rapporteur observed the lack of capacity by the Ghanan correctional facilities to deal with mental illness and the critical shortage of community based mental health services [86]. In contrast, in 2017 two countries were reported to be close to meeting international norms for detention of young people in Rwanda and Mauritania [44, 45].

## Discussion

The scoping review represents a unique and first step toward mapping available literature on the situation of incarcerated or detained young people in the SSA penal institutions. It focuses on an important topic, namely a vulnerable prison population which is at high risk for experiencing violation of their rights. We have presented a broad overview for experts and authorities in the field. Its contribution to the field is twofold, one it summarises and highlights the extraordinarily poor conditions of young people in detention in SSA and second, it draws attention to what is still a clear lack of specific evidence and attention being paid to this issue. We recognise the limitations of this review centring on the relative lack of data sources with only 37 countries represented. Strengths centre on the thoroughness of the review approach in terms of its multi layered strategies to locate all forms of information.

The review highlights that incarcerated or detained young people are a hidden population in SSA prisons who continue to be ignored compared to the adult population in terms of basic conditions such as space, ventilation, sanitation, clothing and nutrition, their personal safety, protection from infectious disease exposure and sexual violence, and their distinct developmental and medical needs. Whilst, they endure the same inhuman, overcrowded and unhygienic conditions as their adult counterparts, the exposure to adult environments and related risk compounds their vulnerabilities to violence and adverse health outcomes [39–41, 43–50, 52–61]. Despite legal mandates that young people should only be detained as a last resort, for the shortest appropriate time and separate from adults, studies found in this review indicate the widespread routine juvenile incarceration with adults, and for lengthy pre-trial periods [29, 35–37, 41, 42, 45, 47–50, 52–55, 67, 68, 74–77, 79, 80, 82, 84, 86, 87]. Young people were not housed with the adult population in only three SSA countries namely; South Africa, Mali and Equatorial Guinea [38, 46, 57, 64, 72].

Emerging from this review is that young people in SSA are incarcerated or detained in situations which do not comply with a host of international UN mandates [38] and specifically the African mandates and agreements such as the African Charter on Human and People’s Rights (1981) [17]; the African Charter on the Rights and Welfare of the Child (1999) [18], and the Southern African Development Community (SADC) Minimum Standards for HIV in Prisons. SSA prison systems are almost universally under resourced leading to deplorable environmental conditions, sanitation and supplies for youth inmates and all inmates A lack of basic sanitation, hygiene and ventilation, inadequate nutrition, clothing, bedding, sheets, blankets and mosquito nets was reported with young people who at times slept on bare floors [28, 34, 35, 40, 41, 45, 48, 63, 65, 67, 68, 70, 71, 73, 74, 78–80]. The situation as for adult prisoners is driven by high rates of pre-trial detention, poor prison infrastructure and a lack of governmental resource allocation. Such factors exacerbate the spread of diseases such as HIV and TB, STIs, body lice, scabies or other skin infections, respiratory, gastro-intestinal and malnutrition related illnesses and deaths [29, 31, 37, 38, 69, 77, 89].

Youth are particularly affected by under resourcing when it means that they are co-housed with adults. Vulnerability of youth in SSA prisons was observed due to lack of personal or family support resulting in a lack of food and basic necessities. This renders vulnerable to exploitation by wealthier and more powerful adult inmates who may prey on them sexually. Hence, the mixing of young people with the adult population increases juvenile risk vulnerability to extreme physical and sexual violence and manipulation, and with that, heightened exposure to HIV and other STIs [37–39, 88]. HIV in SSA prisons is underpinned by a high rate of HIV prevalence on committal, and with certain risk behaviours such as unprotected sex (due to lack of condom provision), injecting drug use and tattooing contributing to HIV spread. This remains a serious public health and human rights issue [31, 36, 38, 90]. This occurs against the backdrop that the SSA region continues to experience a HIV epidemic, with two-thirds $$ \left(\raisebox{1ex}{$2$}\!\left/ \!\raisebox{-1ex}{$3$}\right.\right) $$ of all people infected with HIV living in this region, and with prisoners and young people indicated as particular risk populations for HIV acquisition and transmission, and co-infection with TB [29, 91, 92]. Young people are at high risk of being put in situations where they feel the need to trade sex for basic necessities as evidenced by this scoping review, as well as being exposed to physical and sexual abuse perpetrated by police, prison officers and adult prisoners [29, 36, 38, 39, 58, 60, 61, 71, 75, 79–81, 85].

Like all persons, prisoners are entitled to enjoy the highest attainable standard of health and humane treatment. This right is guaranteed under international law [93–96]. Juvenile health needs and health rights when incarcerated or detained in the SSA prison system and particularly relating to HIV and TB (co) infection have received minimal attention [26, 27, 97]. At present the lack of attention to, and lack of evidence about young peoples’ conditions in SSA prisons contributes to their hidden vulnerabilities. The potential calls for enhanced prison conditions for young people are liable to be integrated into general calls for greater prison resourcing, rather than their unique stand alone needs. Despite agreed international norms in the Standard Minimum Rules for the Treatment of Prisoners (‘Nelson Mandela Rules’) (A/RES/70/175) [8], basic minimum package of health care, or indeed the United Nations Office on Drugs and Crime comprehensive package of HIV prevention, treatment and care in prisons [98–102], the provisions in most penal institutions in SSA are inadequate and in some SSA countries described as alarmingly poor [74]. Access to HIV testing and counselling and to HIV prevention, testing and care (PTC) programmes is often poor in SSA prisons and other closed settings [29]. Of concern is the low resource allocation by government to prison health systems, characterised by shortages of qualified and trained staff, required medical supplies and equipment, and essential medicines [31]. In terms of tackling spread of HIV within prisons, structural barriers which include laws criminalizing “sodomy,” policies or practices limiting bail, and justice system problems resulting in long delays in accessing courts, impede HIV prevention efforts and compound the provision of adequate healthcare for at-risk young people [36, 89, 103, 104]. Despite availability and in some instance low quality availability of counselling services that included SRH, and HIV testing and care, the situation is particularly adverse for young people with low HIV literacy, low uptake of HIV testing services, and who are competing against adult inmates for medical access and care whilst in prison [85]. This has severe public health repercussions for the community upon their return to their homes and families.

## Conclusion

Children and young people should be detained only as a last resort, for as short a period as possible, and separate from adults. Basic rights for incarcerated or detained young people, as enshrined in the UN Convention on the Rights of the Child continue to remain neglected or abused in the SSA region. Children and young peoples international human rights norms are violated in the various forms of abuse illustrated by this scoping review on SSA prisons and youth detention centres. This review highlights the need for enhanced resource allocation to protect young people’s health rights when incarcerated or detained in SSA prisons, alongside the gathering of strategic information and investment in research and gathering of strategic information to inform policy and practice in SSA prisons at country level [29, 31]. Prison authorities have a duty of care to all prisoners in ensuring equivalence of HIV PTC and SRH services for young people detained in prisons, and consistent with international, regional and national human rights standards. The 2016 WHO guiding HIV PTC principles [30] is underpinned by human rights, access to quality healthcare without discrimination, access to justice, acceptability of services, health and HIV literacy and integrated service provision to address multiple (co) infections and co-morbidities. All interventions should be offered voluntarily within an enabling prison environment supported by legislation, policies and strategies, without discrimination based on age, gender, sexual orientation, sexual behaviour, citizenship, country of origin, race/ethnicity, asylum seeking status, religion and substance use status [105–107]. This review highlights the need for continued international technical assistance to countries in the SSA region to support policy reform, infrastructural improvement, and dedicated juvenile and health polices to support those incarcerated or detained as children or young people.

## Additional file

Additional file 1: Supplemental Table. (DOCX 120 kb)

## Abbreviations

ACHPR: African Commission on Human and Peoples Rights
ACRWC: African Charter on the Rights and Welfare of the Child
CCPCJ: Commission on Crime Prevention and Criminal Justice
HEP: Health education and promotion
HIV: Human immunodeficiency virus
HPTC: Prevention, testing and care
IEC: Information, communication and education
MRC: Medical Research Council
NGO: Non-governmental organizations
OHCHR: Office of the High Commissioner for Human Rights (United Nations)
SADC: Southern African Development Community
SDG: Sustainable Development Goals
SRH: Sexual and reproductive health
SSA: sub-Saharan African
STI: Sexually transmitted infection
TB: Tuberculosis
UN: United Nations
UNICEF: United Nations Children’s Fund
US: United States
WHO: World Health Organization
