# Supplementary material for: Prison health situation and health rights of young people incarcerated in sub-Saharan African prisons and detention centres: a scoping review of extant literature
Source: BMC Int Health Hum Rights. 2019 May 22;19:17. doi: 10.1186/s12914-019-0200-z (PMC6532240; doi:10.1186/s12914-019-0200-z)
Supplement: Supplementary file 1 — Supplemental Table. (DOCX 120 kb) [file 12914_2019_200_MOESM1_ESM.docx]

**Supplemental Table**

| **Authors** | **Title, Journal, Year of Publication, Volume, Pages** | **Aim** | **Location** | **Method of Study** | **Results** | **Conclusion** |
| --- | --- | --- | --- | --- | --- | --- |
| **Journals** | | | | | | |
| Todrys KW, Amon JJ, Malembeka G, Clayton M | Imprisoned and imperiled: access to HIV and TB prevention and treatment, and denial of human rights, in Zambian prisons. J Int AIDS Soc. 2011; 14:8. | To better understand the relationship between prison conditions, the criminal justice system, and HIV and TB in Zambian prisons | Zambia | Mixed method  facility assessments and in-depth  interviews with 246 prisoners and 30 prison officers at 6 Zambian prisons; a review of Zambian legislation and  policy governing prisons and the criminal justice system; and 46 key informant interviews with government and  non-governmental organization officials and representatives of international agencies and do | More than 40 inmates reported that sexual activity between male inmates was common, including rape, consensual sex between adults, and relationships with sex traded by the most vulnerable, especially juveniles, in exchange for protection, food, soap and other basic necessities not provided by the prison.  Several prison officers denied the occurrence of sexual activity although others admitted that it occurs  Zambian policy acknowledges***, “Prison confinement can increase vulnerability to HIV due to frequent unprotected sex in the form of rape, non-availability and non-use of condoms, as well as high prevalence of STIs”***  Ventilation in all cells in all six prisons was limited to small windows, and prisoners were frequently confined to their cells for 14 hours each night.  According to both inmates and prison officials, drinking water in prisons is scarce and sometimes unpotable  Food is inadequate, and prison officers reported malnutrition related illnesses and deaths  Routine TB screening is not being conducted and isolation facilities described as pathetic  Government underfunding of prisons resulted in prisoners facing inhuman conditions, human rights abuses, and inadequate access to HIV and TB prevention, treatment or care  Inadequate essential medicines with some health facilities reportedly distributing paracetamol only  Both staff and prison officers indicated that in prisons without in house medical clinics more serious medical conditions requiring advanced care access to care is frequently controlled by medically unqualified and untrained prison officers who evaluate and determine if medical visits to community health facilities are necessary.  Delayed access to higher levels of care was attributed to lack of sufficient prison staff, transportation and fuel by prison officers.  Other barriers to higher levels of care reported included security fears, lengthy delays in the transfer of sick prisoners to medical care outside, in some cases for days or weeks after they fall ill. | Unprotected anal sex in prisons was exacerbated by overcrowding that increased risk vulnerability to transmission and acquisition of HIV among juveniles. Non-availability of basic necessities pushed juveniles to exchange sex for food and soap. The need for the government to avail adequate resources has become urgent and cannot be overemphasised especially in the face of SSA countries experiencing a dual epidemic of HIV and TB. Both conditions are public health communicable diseases known to thrive in overcrowded environments. |
| Todrys KW, Amon JJ. | Human Rights and Health among Juvenile Prisoners in Zambia  Int J Prison Health. 2011; 7(1):10-17 | To explore juveniles experiences of incarceration identify salient issues and probe specific findings raised in the research. | Zambia | Mixed-method study of conditions in Zambian prisons. key informant interviews with government and non-government (NGO) officials, a law and policy review, and interviews with prison officials, researchers conducted a brief prisoner survey and longer, semi-structured in-depth interviews in six Zambian prisons.  246 prisoners, 30 prison officers, and 46 key informants from six prisons were interviewed  Review of, national legislation and policy governing the administration of the prison and criminal justice systems were reviewed. | Hygiene and sanitation for juvenile and adult inmates alike was reportedly inadequate, and basic necessities frequently lacking.  As one 17-year-old, who was wearing rags, told interviewers, *“I have no proper clothes”*  In all six of the prisons visited children were universally held in cells with adult inmates.  One teenage detainee reported being threatened by other inmates (at the instigation of the officers) if he revealed the combined sleeping arrangements  Cells are severely overcrowded, and juveniles were reported to be sleeping three or more to a mattress, with the congestion leading to significant discomfort and the spread of rashes  Food was reportedly insufficient and of poor quality.  Female juvenile inmates did not report specific HIV risk behaviours, while male juvenile inmates reported that they were frequently forced into sexual relationships constituting rape, particularly when held with adult prisoners.  Male juvenile detainees reported that sex was frequently exchanged by juvenile inmates for food and other basic necessities including soap.  An example of the vulnerability of juveniles’ confinement with adult inmates was illustrated at the time of researchers’ visit to one prison, where three juveniles were held in a cell with three adults – two of whom were in prison on charges of defilement of a minor. Condoms were universally unavailable.  When juveniles were asked how they would feel about the availability of condoms ranged from permissive to non-committal  Like adult prisoners, children detained as juvenile inmates frequently are confronted by restrictions on their ability to access medical care.  In all prisons visited, four percent of juveniles had been tested for TB compared with 25% of adults, and 44% of juveniles had been tested for HIV compared with 59% of adults | The failure by the government to provide accommodation that separates juveniles from adults predisposed them to sexual violence that heightened risk to HIV infection. Furthermore, attitudes of prison officers, lack of medical care inadequate staffing and medicines were barriers to access to health care. |
| Kumwenda, M  Nzala S and Zulu J M. | Health care needs assessment among adolescents in correctional institutions in Zambia: an ethical analysis  Health Services Research (2017) 17:581 DOI 10.1186/s12913-017-2532-5 | To explore the situation of health needs among juvenile | Zambia | Qualitative case study design Document review annual reports, quarterly reports, and residential case statistical form reports  Key informant interviews (6)  Two focus discussions and two key informants with  with 35 adolescents, focus group discussions and in-depth interviews were used to collect data. | Beneficence and or well-being for the adolescents was promoted through implementation of health promotion and preventive (HEP) services within the correctional institution  Health promotion activities included awareness talks on health personal hygiene and counselling on STIs with the emphasis on HIV/AIDS and Syphilis.  HEP talks were being by un qualified personnel relying on their experiences to teach  Based on need and on request staff conducted counselling to the juveniles.  Barriers to health awareness raising was lack of comprehensive health care needs assessment.  Nakambala Approved School for juveniles did not have a health facility for treatment of minor ailments  All adolescents regardless of the severity of the illnesses were referred to a nearby clinic.  At the nearby clinic medical, drug supplies and shortage of staff were reported all of which affected provision of treatment and care to the juveniles or adolescent  Sometimes lack of appropriate medicines for recommended standard management of various conditions were observed  There are no health workers attached to the Nakambala Approved School  Follow up of juveniles who had been to the clinic was not conducted by qualified health personnel  There was no continuous close monitoring of ill juveniles because the staff from the clinic did not follow up the juveniles at the school  Lack of adolescent friendly facility at the referral clinic  Non-availability of such services compromised adolescent’s right to privacy and autonomy especially in cases where they had sensitive health issues such as HIV and STIs  Some juveniles were uncomfortable mixing with adults in the reproductive health centre and this was attributed to some cultural values and religious beliefs that stigmatize sexuality among adolescents.  Upon arrival at the institution screening examination was conducted by unqualified staff through observation and history taking  Another key finding was the prevalence of STIs among some juveniles that they had contracted through sexual abuse by adults during remand and in prison  Lack of information on SRH matters affected informed decision making on protected sex.  No health information management system was in place as there were no records of juveniles attended to which hindered referral process and follow ups of the health care challenges or needs among the adolescents.  Environmental factors contributed to high disease burden among the juveniles e.g. prevalence of malaria because the school was located near the largest sugar plantation and stagnant water from irrigation activities  TB was closely linked to HIV/AIDS cases, low body immunity due to poor diet, general poor health and confinement in poor ventilated places.  Skin rashes were common among the juveniles due to the practice of exchange of clothes and beddings in the hostels and the communal use of bathing towels such as sacks (improvised towels) and bathing soap.  Lack of financial resources on the part of the institution as there was no budget line for health care needs assessment. | The lack of a comprehensive package of health care affected the well -being of the juveniles. At this stage of development youth experiment with sex and predispose themselves to a myriad of health problems including STIs and skin conditions hence the importance of screening each and every juvenile on admission which unfortunately was not being conducted Lack of funding has resulted in implementing awareness interventions that are not evidence based. Youth regardless of their station in life, need evidence-based health awareness and promotion activities that are well targeted for them to make informed decisions about their health. . |
| Topp SM, Moonga CN, Luo N, Kaingu M, Chileshe C, Magwende G, Heymann SJ, Henostroza G | Exploring the drivers of health and healthcare access in Zambian prisons: a health systems approach. Health Policy Plan.  HPP. 2016; 31:1250–61. | To identify major structural barriers to strengthening the prison health systems | Zambia | A case-based analysis study using a mixed method approach and drawing on key informant interviews (n=7), memos generated during workshops (n=4) document review and investigator experience. | Some facilities reportedly did not have separate holding facilities for children and transfer to the one (already overcrowded) juvenile facility was observed to take time  Access to a social worker was supposed to be through relevant government department but processes to be followed for one to gain initial access were not clear including follow up mechanisms  Inadequate resources resulting coerced trade in goods and services that increased risk vulnerability to abuse of young people | The mixing of adults and young people in same accommodation exposed the young inmates to abuse heightening their risk vulnerability to HIV infection and other sexually transmitted infections. |
| Lorizzo T | Prison reforms in Mozambique fail to touch the ground: Assessing the experience of pre-trial detainees in Maputo. SA Crime Q. 2012; 2012:29–38. | To explore the conditions of detention and  access to legal representation of 20 pre-trial  detainees in the Central and Civil Prisons of  Maputo. | Mozambique | Qualitative utilizing private face-to-face interviews with the  detainees. The participants were selected by the  authorities of each prison. Open-ended  questionnaires. Numbers not provided | In the Central Prison juveniles were detained with adults but in separate cells in the Civil Prison.  Both prisons are characterised by old and degrading infrastructure. ‘*As paredes estâo cansadas’* [The walls are tired], said one of the detainees interviewed in the Civil Prison.  Walls were cracked but roofs were not leaking,  In both institutions, a health ward opened from Monday to Friday, from 09h00 until 15h00. The service worked as a pharmacy rather than a health care centre  The doctor reportedly gave only Paracetamol for all the diseases you have. If one gets sick after three o’ clock in the afternoon one had to wait until the next morning and nothing is going to change because the only thing, he is going to give is Paracetamol. A prisoner needs to pray God to not get sick from Friday until Monday  Transportation to the civil hospital of Maputo was rare and only happened when the detainee was grievously unwell, as the transfer of sick detainees is seen to increase the possibility of escape  Although access to health care in prison was restricted, this needs to be seen in context since 40% of the Mozambican population has no access to medical services. There are only three doctors and 21 nurses for every 100 000 people in Mozambique. There are approximately 600 doctors in the country  In the Central Prison detainees were sometimes tested for malaria and HIV/AIDS and this was not done on a consistent basis | Just like the adult inmates, juveniles also had to endure unhealthy living conditions and associated challenges. Availability and accessibility of health services in prisons mirrored services available for the general population. However, the general population had an added advantage of being able to seek health services from other health providers unlike in prison settings. Supply chain management of medicines, including human and equipment resources should be addressed as a matter of urgency as per different protocols that the country has ratified. |
| Simooya, O O.; Sanjobo, N E.; Kaetano, L; Sijumbila, G; Munkonze, F H.; Tailoka, F; Musonda, R | Behind walls’: a study of HIV risk behaviours and seroprevalence in prisons in Zambia AIDS 15(13), 7 2001, pp 1741-1744 | To assess the risk of HIV transmission through sex between men, past exposure to sexually transmitted infections, injecting drug use, tattooing and the sharing of shaving equipment | Zambia | Semi-structured questionnaire. focus group discussion with inmates and staff and taking samples from 1596 inmates to test for HIV | Among inmates aged 20 years and below, the HIV prevalence rate was 14.5%  Only 3.9% of inmates reported having sex with other men, but the researchers speculated the figures may be much higher.  Over 50% of men indicated that many were involved in sexual relations, and 6% said that almost all were involved in penetrative sex with other men in prison  In Zambian prisons condoms are not distributed because it is believed that their distribution would encourage homosexuality | In view of the high prevalence of juveniles within the prisons and the poor prison conditions and mixing of adults and juveniles, other non -custodial alternative sentences should be considered by the authorities |
| Sarkin J. | Prisons in Africa: an evaluation from a human rights perspective. Sur Rev Int Direitos Hum. 2008; 5:22–51. | To evaluate prisons in Africa from a Human Rights Perspective. | Africa | Literature review | Most African prison systems with the exception of South Africa, Côte d’Ivoire, Mali, and Angola lack the resources to house children separately from the adult male population  Children imprisoned with the general population have to compete with adults for scarce resources such as food  African prisons fail to meet even the most basic minimum standards for adults hence also fall far short of meeting international standards for juvenile detention.  For example, overcrowding compromises child prisoners’ health and hygiene and exposes them to increased risk of sexual abuse. Juvenile detainees’educational, developmental, health, and nutritional needs are not met | While promising practices have been identified within SSA in relation to juvenile incarceration, most countries however, due to limited accommodation space continue to mix adults and juveniles. Prisons are already under resourced and the needs of these young people are therefore not met. Human rights organizations should monitor prisons and hold to account governments to ensure that they abide to international and regional protocols they have signed. |
| Stout, B | The treatment of children in custody in Lesotho. Article 40. The dynamics of youth justice and the convention of the rights of the child in South Africa.  <http://www.cyc-net.org/cyc-online/cycol-0603-lesotho.html>  2001. Accessed 29 Oct 2018 | To investigate treatment of child offenders in her home country of Lesotho. | Lesotho | Qualitative research semi-structured interviews with 19 children detained in the Juvenile training centre | Ailments that children normally suffered from included coughing, fever and stomach ache  They attributed colds to lack of appropriate clothing. One of the children described the blankets and jerseys that they were provided with as "dilapidated".  The centre had a clinic with a doctor, a nurse and a pharmacist  Overcrowding with between seven and 15 other inmates.  They described their mattresses as torn, the walls as dirty and the floors as dilapidated and cells not warm in winter  Bathed cold water in winter  Poorly prepared food and diet monotonous  Poor sanitary conditions the toilet, bath and washing basin for dishes are located in the same place. | Although the juveniles were held at Juvenile centre they also experienced overcrowding conditions, inadequate bedding and poor quality and quantity of food. The Nelson Mandela Rules and Standard Minimum Rules should be inform the care given to these young people while at these correctional centres. |
| Telisinghe L, Charalambous S, Topp SM, Herce ME, Hoffmann CJ, Barron P et al | HIV and tuberculosis in prisons in sub-Saharan Africa  The Lancet. 2016; 388:1215–27. | To investigate the epidemiology of HIV and tuberculosis in prison populations, describe services available and challenges to service delivery, and identify priority areas for programmatically relevant research in sub-Saharan African prisons. | 24 of 49 countries making SSA | Literature review and case studies in 5 SSA countries | Zambia has no dedicated juvenile justice system, and children in conﬂict with the law face trial in the adult court system  Children held with adults often face sexual violence***.***  Juveniles end up either taken advantage of or enticed because of their vulnerability.  Access to health care, that is often diﬃcult for adults, is equally difficult for juveniles.  All South African prisons have internal clinics but these clinics are understaﬀed.  A nursing act that prevents nurses prescribing without authorisation contribute to time lags and bottlenecks in chronic-disease management  Increased dependence on non-governmental organisations to deliver tuberculosis and HIV testing and treatment  Prevention services are weak because nurses frequently are not adequately trained in primary care or preventive health  Notable dearth of sex-disaggregated and age-disaggregated data describing HIV or tuberculosis treatment outcomes for women and children within sub-Saharan African prisons | Mixing juveniles with adult inmates increased risk of violence towards the young people. Barriers to accessing to health were attributed to negative attitudes by prison officers. Prison Health is good public health. Urgent retraining and re-orientation of prison officers is needed to impart them on rights approach to service provision for juveniles. There is an urgent need for effective and efficient Health Management information systems in prisons in sub-Saharan Africa both for surveillance purposes, policy formulation and resource allocation. |
| James G | Assessment of the Administration and Practice of Juvenile Justice System in Abuja, Nigeria  MJSS.4 (1) 331-338 | To examine the practice of juvenile justice system in Nigeria, with the view to assessing the effort of government in the administration and control of juvenile delinquency in line with established standard in Federal Capital Territory, Abuja | Nigeria | Cross sectional study using structured questionnaires to elicit information from the respondents that included Police, Prison Service and the Judiciary n=400 | Two-thirds of the respondents agreed that juveniles are subjected to poor conditions in police and prison cells, subjected to verbal and physical assault, and are poorly fed  Half agreed that they are not separated from adult prisoners.  overwhelming majority (74.7%) revealed that the prison cells where juveniles are kept, along- side with adults are congested.  Two-thirds of the respondents agreed that there are no adequate feeding and medical care as well as accommodation and sleeping materials in the juvenile institutions.  The institutions are inadequately funded; lack qualified and competent staff, as well as counselling services. | Just like other juveniles in SSA prisons, juveniles in this Nigerian prison also faced a myriad of challenges. Inadequate funding by the government affects the care juveniles get in custody. It is imperative for juveniles to have access to counselling services given their developmental stage characterised by lots of challenges that range from social, psychological to health related. |
| Bella1, T T; Atilola, O and Omigbodun OO | Children within the juvenile justice system in Nigeria: psychopathology and psychosocial needs  Ann Ibd. Pg. Med. 2010 8;34-39 | To identify the psychosocial needs as well as types of psychopathology among a group of incarcerated children at the Ibadan remand home. | Nigeria | A cross-sectional survey of children and adolescents at the Ibadan remand home was carried out using a semi-structured questionnaire. | 90% were in need of care and protection.  All had significant psychosocial needs presenting as difficulty with their primary support, economic, social environment, or educational systems.  The majority (97%) demonstrated significant psychopathology and anxiety, suicidal and depressive symptoms were the most commonly elicited.  Meagre health services are provided by the nurses who work few hours between 8 – 4 pm on weekdays only  No provision for emergency services and the home lacks even a first-aid box. | The unconducive prison environment is bound to bear down on the imprisoned juveniles resulting in stress and depression. Therefore, an urgent need arises for supportive psychosocial services to help them cope with the challenging prison environment. |
|  | Centre for the Study of Violence and Reconciliation (CSVR). Doing time in a Gauteng Juvenile Correctional Centre for Males. CSVR, Gauteng, 2004  <https://www.csvr.org.za/docs/gender/doingtime.pdf>  Accessed 20 Nov 2018 | To explore male youths’ experiences of violence, sex and sexual violence in prison as well as on more general personal features and situations in the institution of the Boksburg Youth Correctional Centre (BYC). | South Africa | Survey using face-to-face interviews with 311 randomly selected participants. | Most of the young men (91%) were staying in communal cells where the recommended capacity for these cells was 30–40 people. They were sharing with 26–35 other people indicating that they were (mainly) not being held in overcrowded conditions.  Visitors regularly represent a key source of both basic necessities and luxuries for offenders.  Access basic necessities is also an important facet of inmate power structures and those without access are often amongst the most vulnerable to these structures and the hardships of prison.  Twenty-two percent said they didn’t receive anything from outside, while money, toiletries and clothing were the items most likely to have been received by those who did receive things from outside  8% of those who had served less than a year of their sentence estimated that prison had provided them with most of their information about sex.  Nearly all respondents (99%) said they had heard of HIV and AIDS.  Most had also received some form of HIV and AIDS information whilst in BYC  43% respondents had received HIV and AIDS information via a programme or counselling service offered by a DCS member  5%of participants had had sexual intercourse in prison in the last six months, and 5% had had other types of sex (but not intercourse) such as thigh sex, oral sex or mutual masturbation  Those having sex in prison tended to have either “other kinds” of sex or intercourse as well as “other kinds” of sex rather than sexual intercourse exclusively.  6%of respondents had, during their time in BYC, had “casual sex”. This was defined as “sex with someone once or a few times without paying or being paid anything, and without forcing or being forced to have it”. In all but one case - where it had been with a correctional officer - casual sex had taken place with another inmate.  5% of respondents had, during their time in BYC, been in a “mutually agreed relationship” defined as where “neither one… was being forced or paid to be in this relationship”.  These relationships were with other inmates, except in one case where the respondent had been in relationships with both staff member(s) and inmate(s). | The South African model of accommodating juveniles could be used as promising practice within the SSA region and replicated in other countries within the region. |
| Atilola, O | Juvenile/Youth Justice Management in Nigeria: Making a Case for Diversion Programmes. CCJ 2013(1) 3-16 | To examine s article examines how the lack of well-established alternatives to child/youth incarceration in Nigeria leads to human rights violations. | Nigeria | Document review | Overcrowding in the borstal homes  Mixing of young people with adults with 30 children aged 12 years reportedly held together with adult prisoners in one prison in Abuja  The number of borstal homes (or young people prisons) was inadequate in terms of number, spread and gender equality. | The challenge of overcrowding and mixing of young prisoners with adults is a common phenomenon across most SSA a situation which increases disease vulnerability of young prisoners to abuse. |
| Media Reports | | | | | | |
|  | Prisons ignoring the rights of Ivorian youth [http://www.irinnews.org/analysis/2015/05/21/prisons-ignoring-rights-ivorian-youth Accessed 1Nov 2018](http://www.irinnews.org/analysis/2015/05/21/prisons-ignoring-rights-ivorian-youth%20Accessed%201Nov%202018) | To document experiences of the country’s justice systems from different perspectives | Côte d'Ivoire | Qualitative  Interviews with juvenile prisoners and Magistrate, representative for UN UNICEF, senior official at Cote d’Ivoire’s National Commission for Human Rights, Ministry of Justice’s Judicial Protection of Children program, prison guards | Hundreds of children are being kept behind bars in Cote d’Ivoire’s overcrowded adult prisons waiting on trial dates due to the country’s broken post-crisis criminal justice system.  Cases of abuse by adults who can easily access the cells where minors are kept, which themselves are practically in the same detention facilities as adults.”  The conditions of detention are not satisfactory at all and certainly not in line with the international standards, [which require] the complete separation of adults and children  The cells are cramped; the toilets non-functional; and there is no running water for bathing.  Lack of hygiene, backed by inadequate medical treatment, has meant many of the young prisoners are in ill health.  Most of the young prisoners that IRIN spoke to said they slept on the floor because there were inadequate mattresses or bedding to go around.  Only a handful of torn mosquito nets were available to protect them against malaria and visits from their parents or other family members were often denied for no reason. | The practice of keeping the juveniles in the same detention facilities with adults compromises their security. The government should improve conditions of detention for the juveniles and conditions should be in line with minimum standard rules (MSRs and Nelson Mandela Rules (NMRs) which are recognized in international law |
| **United Nations Joint Programme on HIV/AIDS (UNAIDS) United Nations Children's Fund (UNICEF), United Nations Office of Drugs and Crime (UNODC), United Nations Political Office for Somalia (UNPOS)** | | | | | | |
|  | Assessment of the Prison System in Mogadishu South Available from:  <https://unpos.unmissions.org/> Accessed 24 May 2018 | To assess the prisons sector in South Central Somalia/Mogadishu. | Somalia | Inspection and qualitative interviews  Series of visits to the Mogadishu Central Prison and meetings with Government officials from various institutions within the criminal justice sector, including representatives from the Ministry of Justice, Religious Affairs and Endowment, Custodial Corps, and the Judiciary. Consultations with representatives of the Civil Society and interviews with prisoners detained at the Mogadishu Central Prison. | There are no separate specialized young offender`s institutions for juveniles and all minors committed to prison are sent to the adults’ 33 prisons  A lack of separate facilities for girls. This situation poses a serious threat for both the physical and psychological wellbeing of children in detention.  Juveniles in detention and/or prisons also do not have access to psychologists or social workers  Overall, it is clear the conditions for juveniles in prison are inhumane and in breach of the conventions  Medical care lacking  Shortage of equipment and staff  No soap  Lack of access to clean water and adequate sanitation facilities | The need for psychological counselling for the juveniles incarcerated under such inhuman and degrading conditions cannot be over emphasized These juveniles still have a life ahead of them despite being in prison. They still have the ability to meaningfully contribute to the development of society provided they are given the right conditions to reform. Inhumane degrading treatment and conditions in correctional facilities is likely to harden them instead of instilling correctional behaviour for the time they are incarcerated. |
|  | Opening minds to rights behind bars. Report into the situation of detention in Sierra Leone.  <https://unipsil.unmissions.org/sites/default/files/behind_bars.pdf>  Accessed 29 Nov 2018 | To support the government of Sierra Leon in fulfilling its responsibilities towards people in detention through assessing effectiveness of technical assistance and capacity building interventions its implications on the corrections system | Sierra Leone | Systemic review of the status definition in Sierra Leone in 17 prisons and 3 juvenile detention centres through monitoring visits using a checklist. | The report [documented dilapidated infrastructure](http://unipsil.unmissions.org/LinkClick.aspx?fileticket=KYcKmiqbe6E%3d&tabid=9621&language=en-US) and inadequate maintenance.  Insufficient bedding for inmates  Prison cells were described as often lacking proper lighting, bedding, ventilation, and protection from mosquitoes  Limited and poor medical facilities  Access to food and drinking water was often inadequate.  Juvenile detainees were sometimes mixed with the adult prison population increasing the risk of abuse and exploitation  Detention conditions remained below minimum international standards because of overcrowding, unhygienic conditions, and insufficient medical attention.  Most prisons visited did not have piped water systems, and some prisoners lacked sufficient access to drinking water | Most of the penal institutions were built during the colonial era and in need of a face lift. Just like in other SSA countries conditions of incarceration were found to be in a dire state and exposed both` juveniles and adults to disease. |
|  | United Nations (UN) General Assembly. Report of the Special Rapporteur on torture and other cruel, inhuman or degrading treatment or punishment, Mission to Ghana. United Nations, New York. 2014. <https://www.ohchr.org/en/issues/torture/srtorture/pages/srtortureindex.aspx>.Accessed 28 Apr 2018 | To conduct an independent and objective scrutiny of Ghana’s human rights situation, particularly in regard to a number of critical issues in its criminal justice system and mental health-care practices. | Ghana | Meetings with  representatives of the Ministry of Foreign Affairs, the Ministry of the Interior, the Ministry of Justice and the Office of the Attorney–General, the chief psychiatrist of the Ministry of Health, members of the Ghana Police and Prisons Services, the Military Legal Directorate and the judiciary, and the Commissioner of the national human rights institution of Ghana, the Commission on Human Rights and Administrative Justice and representatives of United Nations agencies and non-governmental organizations (NGOs). | Juveniles may only be convicted by a juvenile court and the Senior Correctional Facility in Accra, with a capacity of 300, is the only juvenile facility in the country.  At the time of the visit by the Special Rapporteur there were 100 boys aged between 12 and 18 at the facility.  The centre also offers vocational training in 15 different workshops, including on information and communications technology.  The facility is an open camp with communal houses for sleeping. No complaints regarding the food and, as described, meals seemed better in comparison to what is provided in adult prisons.  Medical care is provided by medical aides, not professional doctors or nurses, just as in adult prisons | The availability of better quality and quantity of food is commendable although medical care was provided by non-medically trained staff. There is need to ensure that similar facilities are available countrywide instead of just the one centre in Accra |
|  | Office of the High Commissioner for Human Rights (OHCHR). Special Rapporteur on Eritrea. OHCHR. Geneva, 2014. https://www.ohchr.org/en/hrbodies/sp/countriesmandates/er/pages/sreritrea.aspx Accessed 29 Nov 2018 | To investigate and report on the situation of human rights in Eritrea | Eritrea | Qualitative interviews with different stakeholders including Eritrean refugees during field missions in 2013 and 2014  human rights in Eritrea, by means of interviews with victims of human rights violations Visits to detention centres and prisoners | Although there is a juvenile detention centre in Asmara, children below 18 years, especially those rounded up during giffas, are held with adults in detention centres before being transferred to a military training camp  Severe overcrowding in prisons is a major issue that spawns several other problems relating to the health, hygiene and nutrition of those in custody  80 inmates were sometimes said be held in an underground small cell measuring 10 m by 15 m, with poor ventilation, no windows or light.  The holding cells have no sanitation facilities and prisoners are only allowed out for very short periods to use the toilet.  Personal hygiene is a serious concern with detainees suffering from body lice, scabies or other skin infections, and prone to respiratory complaints or diseases and diarrhoea.  Medical facilities are minimal and detainees with chronic health problems do not have easy access the right kind of medication or treatment, thus endangering their lives.  Referral to hospitals takes time.  Food is of poor nutritional quality and inadequate in quantity, thus exposing those in custody to malnutrition.  Meals invariably consist of bread and lentils and access to drinking water is limited.  Inmates sleep on the floor without proper bedding. | Overcrowded environments have been known to be a major cause of spread of communicable diseases globally in public health. The tuberculosis mycobacterium thrives in overcrowded environments with poor ventilation. SSA has currently two dual epidemics mainly HIV and TB. Other alternatives to detention and incarceration of juveniles should be explored to decongest prisons and safeguard the health of both young and adult offenders alike. |
|  | World Organisation Against Torture (OMCT). Human Rights Violations in Benin.Geneva,2004. [www.omct.org/files/2004/10/2574/report_benin_eng_10_2004.pdf](http://www.omct.org/files/2004/10/2574/report_benin_eng_10_2004.pdf)  Accessed 29 Oct 2018 | To produce an alternative report that is a valuable source of information for the independent experts for analysis of implementation of the United Nations human rights instruments in order to objectively assess government action to eradicate torture and other cruel, inhuman or degrading treatment or punishment | Benin | Document review of human rights organizations reports and interviews but numbers not indicated | Out of the eight prisons existing in Benin, only prison of Parakou does not have special quarters for minors.  A recent visit carried out by ESAM to the prison of Cotonou showed that the conditions of detention for minors are poor.  Problems observed related to inadequate living conditions, poor hygienic conditions, lack of games and leisure, and no medical care.  Between 20 and 30 minors live in a tiny room.  They sleep together on bare ground, apart from the overpopulated adult prison quarters.  Minors in preventive detention are kept with others who have already been convicted,  Some adults are present in the minor quarter on the authorisation of the head of the prison. | Mixing young people that have not yet been sentenced with those sentenced and adults being present in minor quarters should be not be allowed as it plays a major role in shaping the behaviour of the juveniles before they are even sentenced. It also predisposes the minors to abuse between themselves or abuse by adults. |
| **Human Rights Organizations** | | | | | | |
|  | International Child and Youth Network (CYC-Net). The state of juvenile justice in Malawi. Western Cape, 2000.  [http://www.cyc-net.org/cyc-online/cycol-0400-malawi.html Accessed 30 Oct 30 2018](http://www.cyc-net.org/cyc-online/cycol-0400-malawi.html%20Accessed%2030%20Oct%2030%202018) | To review of the legality of the detention of juveniles currently in prison. They examined the juvenile section of three prisons | Malawi | Interviews with 383 inmates and examination of juvenile sections of 3 prisons | Separation from adults: Poorly supervised  Sexual abuse was well documented  Health/ Sanitation: poor | Lack of monitoring and supervision of prison officers by higher management is cause for concern in view of highlighted sexual abuses. Human rights and advocacy organizations should also monitor government |
|  | Amnesty International. Poverty, isolation and ill-treatment. Juvenile justice in Burundi, AFR 16/011/2002. New York, 2002.  https://www.amnesty.org/en/documents/afr16/011/2002/en/  Accessed 30 Oct 2018 | To document the plight of child detainees in the country | Burundi | Visits to six of Burundi’s 11 prisons. Interviews with former and current detainees, national and international human rights groups, including the Association burundaise pour la Défense des Droits des Prisonniers (ABDP), Burundian Association for Prisoners’ Rights, the Association burundaise pour la protection des droits humains et des personnes détenues (APRODH), Burundian Association for the protection of human rights and detainees, the Ligue ITEKA, Burundian Human Rights Ligue, the Office of the United Nations High Commissioner for Human Rights, Avocats sans frontières, Lawyers without Borders, and others working on behalf of child detainees, as well as members of the judiciary, penitentiary services and government officials and juvenile detainees | Some interviewed children spoke of sexual abuse within prison by other prisoners (children are largely detained with adults).  Boy detainees appear to be particularly vulnerable to sexual abuse as girls attain a degree of protection where they are held in women’s wings.  In addition to these abuses, children are also being stifled by lack of activities and education, are malnourished and often entirely without the contact and support of their families  The investigation concluded that 70 per cent of children lived solely on the prison diet, which provided only 1,990 calories instead of the necessary 3,000; not only did children sleep in the same rooms as adults, the rooms were extremely overcrowded; hygiene was poor and access to medical care limited | Although girls had a degree of protection by being held in the women’s wing boys were mixed with adults under extremely overcrowded, unhygienic conditions and limited access to health care. This created a conducive environment for unprotected sexual abuse of the juveniles. |
|  | Commonwealth Human Rights Initiative (CHRI), Africa Office. Juvenile Justice in Ghana. A Study to Assess the Status of Juvenile Justice in Ghana 2011. Commonwealth Human Rights Initiative (CHRI), Africa Office, Accra, 2011. www.humanrightsinitiative.org/publications/ghana/JuvenileJusticeinGhana.pdf  Accessed 1 Nov 2018 | To assess the Status of Juvenile Justice in Ghana | Ghana | Qualitative held meetings with various members of the juvenile justice system in Accra. Feld visits to the Girl’s Correctional Centre, Girl’s Remand Home, and the Boy’s Remand Home. Observations and Review of international and regional statutes | Juveniles are placed with adult offenders  Frequent cases where juveniles are held in the same cell or in the same area as adult offenders  Before the juvenile is tried or sentenced, they will often be sent to one of the remand homes in Osu.  The Girl’s Remand Home is located on the same compound as the Boy’s Remand Home but still separated as required by the Juvenile Justice Act.  The Girl’s Junior Correctional Centre is also located in the same building as the Remand Home.  Social workers and volunteers give mainly social education to the boys at the remand home, on topics such as health, HIV, environment, and drugs .  The social workers give counselling themselves, but they also have the use of a clinical psychologist that comes to the compound once per week. Social workers also counsel the boys along with the clinical psychologist who comes once a week.  The buildings at the compound were built in 1946, and very little has been updated since then and is in . disrepair.  Insufficient space to adequately house juveniles.  Insufficient social workers due to the number of juveniles at the facility and the supervision required | Juvenile boys and girls are not accommodated with adults. Health education is given on HIV and environment and dugs. Despite the inadequacy of social workers, this is the only country giving education on drugs. Health education and awareness on drugs as they affect young people is key to the well-being and choices young people make at this stage of development. Risk taking, peer pressure and experimentation is particularly attractive to juveniles as they are bound to experiment not only with drugs but sex as well, Hence the need to have adequate staffing in terms of social workers and psychologist who should be available as and when need arises. |
|  | American Bar Association, Africa Law Initiative and UNICEF. Assessment of the Liberian juvenile justice system. American Bar Association Africa Bar Initiative. Monrovia, 2006  https://www.oijj.org/en/docs/general/assessment-of-the-liberian-juvenile-justice-system  Accessed 1 Nov 2018 | To assess the Liberian Juvenile Justice System | Liberia | Qualitative with key stakeholders from the Ministry of Justice, the Judiciary, the Juvenile Judge, police officers, prison officers and administrators, representatives of international organizations and non-governmental organizations (NGOs) and child advocates. | Obvious concerns about the conditions of confinement for all of the detainees were raised.  Children detained in tiny cellblocks with 2 to 5 other youth  The food barely edible, and the cell block sizes were unbearably small and too small to allow multiple inmates to sleep lying down at once  Many of the inmates were wearing the same clothes they entered the facility with many months before and there was tremendous overcrowding  Because there were no uniforms for the guards, the demarcation between prisoners and guards was virtually impossible. | In view of the documented negative impact of overcrowding there is an urgent need to improve the infrastructure and ensure consistent adequate supply of basic supplies. The diversity and multiplicity of different organizations working on Juvenile issues need effective coordination to get maximum value out of the different efforts. |
|  | Amnesty international. Chad: ‘We are all dying here'; human rights violations in prisons. Amnesty international. London, 2012. <https://www.amnesty.org/download/Documents/16000/afr200072012en.pdf>  Accessed 1 Nov 2018 | To document the conditions in Chad’s prisons and exposes human rights violations committed inside them. | Chad | Qualitative including desk documents review prison visits, interviews with prison officials and incarcerated prisoners   numbers not given in the report | Prison conditions in Chad are so deplorable that they amount to cruel, inhuman and degrading treatment or punishment  Cells are severely overcrowded, and food and drinking water are inadequate and sometimes not available.  Prisoners are dependent on family and friends to supplement their diet and provide other necessities meaning those without support or held in a prison far from their home, go without.  Children, including young girls, are detained together with adults.  Health care and medical services do not exist in the majority of prisons in Chad.  Amnesty International delegates found that in several prisons many prisoners required medical care and were suffering from skin diseases and rashes, and no treatment was provided.  Lack of medical staff in some prisons had other prisoners with no medical training giving care to peers  Those suffering from serious transmissible diseases such as tuberculosis, or sexually transmitted infections and HIV are particularly at risk. | Juveniles like their adult counter parts endure the same inhabitable prison conditions. Inadequate medical provision and failure to isolate those suffering from infectious diseases such as TB can result in a public health catastrophe due to the emergence of multidrug resistant TB that will spread not only among the inmates but officers and beyond the prison walls into the general population. Surveillance and prison conditions need to be under continuous monitoring to curb the spread of communicable diseases |
|  | Y Care International. Young people in conflict with the law in Togo, West Africa 2014. Y Care International. London, 2014.  <http://www.ycareinternational.org/wp-content/uploads/2015/06/YCI_Young-people-in-conflict-with-the-law-in-Togo_3.pdf>  Accessed 9 Nov 2018 | To establish the current situation for young people detained in Togolese prisons. | Togo | Baseline survey done but no mention of sample size and data collection technique | Over-crowding was reported as a critical issue in Lomé prison  Three quarters of detainees were sharing a cell with over 50 people  95 % of them stated that their cell, which rarely exceeds 6 metres x 5 metres, is too small.  The majority of detainees 70% are in Lomé prison which is over three times its stated capacity.  The Atakpamé prison, which is a much smaller site, had a 175% occupation rate  In Lomé, Atakpamé and Kara prisons, an overwhelming majority of detainees complain of their cell’s poor air circulation.  Conditions in prisons are equally poor and are denounced by Human Rights defenders.  Healthcare standards are alarmingly poor. | Human Rights defenders play a critical role not only in raising awareness on critical issues that affect juvenile prisoners incarcerated in such inhumane and degrading conditions but also advocating for better conditions of incarceration. |
|  | Human Rights Watch (HRW). Unjust and Unhealthy HIV, TB, and Abuse in Zambian Prisons New York, 2010.  <https://www.hrw.org/report/2010/04/27/unjust.../hiv-tb-and-abuse-zambian-prisons>.  Accessed 25 Nov 2018 | To analyse prison health conditions in Zambia by independent human rights organizations. | Zambia | Qualitative interviews with 246 prisoners, eight former prisoners, 30 prison officers  Facility tours | Minors mixed with adults  Share living quarters with other inmates with infectious conditions like TB  Insufficient overflowing toilets and bathing facilities with some water points close sanitation outflows  Bathing buckets sometimes doubling up and used as toilet facilities in the night  Detainees sleeping up to 5 on mattress, dirty unwashed blankets and mattresses full of lice and dust,  Remanded prisoners not provided with uniforms and convicted prisoners’ uniforms grossly inadequate only one pair with no change pair  Food insufficient and of poor quality  Mosquito nets not provided  Non-availability of potable water due to non-payment of water bills by the authorities and water shortages resulted use of unclean water  Access to health services controlled by cell captains who sometimes deny other inmates access  Delays in accessing outside health care due administrative challenges like lack of transport, security fears and negative staff attitudes  Lack of staff and medicines for treatment of minor ailments | Despite the country having ratified international, continental and regional protocols on human rights conditions under which juveniles are kept in Zambian prisons is a cause for serious concern that should be addressed through monitoring by civil rights organizations. A holistic approach to addressing these myriad intertwined challenges should be adopted by both state and non-state actors |
| **African Union Reports** | | | | | | |
|  | African Commission on Human and Peoples’ Rights (ACHPR). Malawi: Mission on Prisons and Conditions of Detention - 2001/Malawi/States/ACHPR. L'Exprimeur, Paris. 2002 <http://www.achpr.org/states/malawi/missions/prisons-2001/>  Accessed 20 Aug 2018 | To assess and document the conditions of detention in Malawi. | Malawi | Consultations, inspections and interviews 13 prisons visited, 4 consultations with authorities, 6 with NGOs, 5 with media houses and prisoners’ interviews in private, individually or in groups in each prison and police station visited. | Homosexual practices primarily target juveniles and other vulnerable prisoners.  At Zomba, juveniles complained that they could be transferred to the adult units by prison officers themselves to be abused by adult prisoners. It was also alleged that young adults were abused by elders.  The delegation observed that the treatment of juveniles was worse off than that of any other prisoners  Juveniles were reportedly suffering more from overcrowding, lack of exercise, lack of educational programmes, lack of legal assistance, harsh sentencing practice, sexual abuse.  In some prisons visited juveniles complained that some of them had been victims of sexual abuse when they were transferred to the adult section of the prison as they reached their maturity and were considered to be old enough to be transferred to this section.  Adults would propose sex in exchange for food and a place to sleep.  Some young prisoners interviewed in Zomba adult sections confirmed that there were many cases of homosexual relations. Adults would help young prisoners but also abuse them and use them as their "wives".  At Zomba, some juvenile prisoners also reported that some officers were paid by adult prisoners to organise the transfer of juveniles to the adult sections.  A report on HIV/AIDS in Malawi prisons commissioned by Penal Reform International (PRI) also documents this problem.  The Special Rapporteur recommended that the Malawi authorities to take up this issue and ensure that juveniles and young prisoners in Malawi are no longer subject to violence and degrading treatment and that their dignity is respected.  Authorities should in particular ensure that separation of adults and juveniles is strictly enforced.  Authorities should also inquire about allegations of transfer of juveniles to adult sections and punish all prison officers who would be party to this terrible traffic | Prison officers are supposed to be the custodians of the young prisoners and not act contrary to international protocols which they should be upholding by exposing young inmates to sexual and physical abuse Human rights organizations and Prison authorities should closely monitor and hold to account any prison officer found to have violated the rights of the young inmates and trafficking them to adult sections to be sexually abused |
|  | African Commission on Human and Peoples’ Rights (ACHPR). Mozambique: Mission on Prisons and Conditions of Detention - 2001/ Mozambique/ States/ACHPR. Gambia, 2001  <http://www.achpr.org/states/mozambique/missions/prisons-2001/>  Accessed 26 Jan 2018 | To assess the implementation of the recommendations made by the Report of the Special Rapporteur in 1997 | Mozambique | Inspections, interviews, meetings observations and document review. eight prisons visited and one police station, Consultations undertaken with prison authorities 16 including contracted physicians, nurses and helpers at places visited, NGOs eight, and prisoners | Young offenders were, detained together with adults and convicted criminals.  Rather than a place of reform, this is more likely to lead to abuse by adults and further criminalisation of the young prisoners.  Overcrowding  Inadequate centres and medical staff  Food and water/sanitation problems.  Hygiene problems due to lack of soap, of cleaning tools, and limited access to bathing facilities and water. | Mixing juvenile prisoners with adults breeds problems such as physical and sexual abuse thereby predisposing the young people to communicable diseases especially sexually transmitted infections. In line with international, continental and regional protocols governments should allocate enough resources to prisons to ensure that the they are adequately funded and resourced. |
|  | African Commission on Human and Peoples’ Rights (ACHPR). Namibia: Mission on Prisons and Conditions of Detention - 2001/Namibia/States/ACHPR  http://www.achpr.org/states/namibia/missions/prisons-2001/  Accessed 26 Jan 2018 | To assess and document the conditions of detention in Namibia. | Namibia | Interviews, Observations and document review of 10 prisons  Consultations with policy makers at various levels and administrators, various stakeholders including NGOs and the media, individual prisoners and of in groups | Young and destitute prisoners agree to pair with adult prisoners in the secret hope that they will see their living conditions improve; other do so in the hope to get more food.  Inadequate food  Inadequate medical and paramedical stuff  Medical clinics not available in all prisons  No counselling services due to lack of trained personnel | Access to basic necessities of life is a human right enshrined in global, continental and regional statutes and conventions to which many countries are signatories. Placing young people in desperate environments where these are not adequately provided for increases their risk vulnerability to abuse. |
|  | African Commission on Human and Peoples’ Rights (ACHPR). Uganda: Mission on Prisons and Conditions of Detention - 2001/Uganda/States/ACHPR. Niger. 2003.  <http://www.achpr.org/states/uganda/missions/prisons-2001/>  Accessed 26 Jan 2018 | To evaluate and document conditions of detention in Uganda | Uganda | Interviews, meetings Observations and document review  13 prisons visited, 6 police stations, 1 remand home for juveniles, discussion conducted with 7 authorities3 NGOs representatives, 9 representatives of media houses and individual prisoners and in groups. | No special court for juveniles.  According to art. 34 (6) of the Constitution: ***"****a child offender who is kept in lawful custody or detention shall be kept separately from adult offenders".*  This was not always the case, particularly for girls  A few juveniles were detained at Luzira, due to lack of screening facilities at Mulago hospital and others at Ihungu local administration prison.  Two very young girls of 15 and 16 years of age were at Kakiika women section.  Juveniles in Luzira Upper prison shared a ward with elderly people, ward leaders and religious leaders.  Some of them had been victims of sexual assaults by other prisoners.  Prison authorities were said to ignore reports of the victims of sexual assaults  Nagguru remand home, housed 134 juveniles, out of which seven were girls but with a capacity of 45 and remand prison was built in 1954 and the youngest juvenile was 12 years old.  Once convicted, juveniles will be transferred to a rehabilitation centre and a number of programmes have been set-up for these juveniles:  Counselling services for new-comers, sick people and those who have spent a long time on remand or the convicted.  Training in life skills such as carpentry, needlework, art, etc. | Overcrowding and making juveniles share accommodation with elderly prisoners predisposed the adolescents to communicable diseases due to sexual abuse. The Justice system should monitor prison authorities and address the issue of sexual abuse as reported by the juveniles |
|  | African Commission on Human and Peoples’ Rights (ACHPR). Cameroon: Prisons and Detention Conditions - 2002/Cameroon/States/ACHPR. Gambia, 2002.  <http://www.achpr.org/states/cameroon/missions/prisons-2002/>  Accessed 26 Jan 2018 | To evaluate and document conditions of detention in Cameroon. | Cameroon | Interviews, meetings observations and document review  8 prisons and 4 police stations visited. Consultations with 24 with authorities, 4 NGOs, 1research institutions and 8 media houses | Generally, men, women and minors are kept separate in prisons, but due to overcrowding, there was no uniformity meaning some juveniles shared accommodation with adults in some prisons  The menu is poor and the quantity inadequate.  Food is not well cooked and falls short of meeting minimum hygienic requirements  Shortage of medical personnel assigned to the various prisons, police stations and gendarmeries  Shortage of the relevant drugs to treat prisoners with inmates sometimes forced to pay for the medical services they are supposed to have access to for free.  A serious problem of TB spreading in the prisons and gendarmeries alike, leading to death  High demand for medical services reported.  Severely ill inmates sometimes have to wait several days before they could be taken to the hospital,  Water is highly scarce and deplorable waste management.  Delay was reported to be due to administrative reasons or lack of transportation.  Some sick inmates were left out in their cells without any medical or other assistance due to overcrowding, which made it conducive for disease spread  Poor hygienic conditions and ventilation  Lack of basic amenities like soaps and toiletries, and the alarming rate of congestion.  There is a strong stench in the various premises. Police cells emit the worst odour (hygiene and toilets)  Sanitation materials are not regularly and adequately issued to inmates.  No Information, Education and Communication (IEC) materials on HIV& AIDS and health education and promotion on sexual reproductive health | Conditions impacting on health service provision to juvenile prisoners are similar to other mission reports and need a holistic approach to addressing them |
|  | African Commission on Human and Peoples’ Rights (ACHPR). Ethiopia: Mission on Prisons and Conditions of Detention - 2004/Ethiopia/States/ACHPR. Gambia, 2004.  http://www.achpr.org/states/ethiopia/missions/prisons-2004/  Accessed 26 Jan 2018 | To monitor prisons and other places of detention in Member States of the African Union | Ethiopia | Qualitative meetings, observations and document review  visited and inspected nine prisons, two prison farms and two police stations. | The prison structure does not adequately take care of juveniles. There is only one juvenile court in the whole country and based in the capital, Addis Ababa.  There are no juvenile facilities in the regions – reformation centers or courts.  Juveniles are accommodated together with adults in all the prisons.  In the Harar Prison, the authorities have allocated a cell for juveniles but at the time of the visit, adults were found going in and out of the cell and it is not completely separated from the adult section.  Prisons poorly staffed with health staff with the exception of Addis Ababa prison  Prisoners not provided with either mattresses or blankets and basic necessities such as soap, sheets, detergents for clothes are equally not provided.  Such items are either provided by NGOs or the Prisoners Committees.  Shortage of water | The lack of juvenile prisons across the country does not augur well for juveniles that are mixed with adults a consistent trend that has emerged in most of SSA prisons including lack of provision of basic necessities from toiletries to food, health care and bedding. NGOs and Prisoner’s committees should be complementing government efforts as it is the responsibility of the state to provide supplies. |
|  | African Commission on Human and Peoples’ Rights (ACHPR). South Africa: Prisons and Detention Conditions - 2004/South Africa/States/ACHPR. Gambia, 2004. <http://www.achpr.org/states/south-africa/missions/prisons-2004/>  Accessed 26 Jan 2018 | To draw the attention of prison officials to the treatment of persons deprived of their liberty. | South Africa | Qualitative and quantitative Inspections, interviews, meetings with national and local prison officials, closed door meetings with detainees, and communications from civil society organisations and information from various organisations i.e. 19 Detention centres, one mental hospital, one, repatriation centres, six prisons, one Child Justice Centre, Prisons six, Police stations four, youth centre one and eight institutions | In the Drakenstein Medium B, there were 680 juveniles engaged in formal education and other informal education such as HIV/AIDS peer education programme and computer training programmes.  St Alban’s Prison, there are 717 juveniles. They are separated from the adults. Those between 18 – 20 years are held in single cells with each cell holding three juveniles.  The cells measure about 3m x 7m and were meant to hold only one juvenile.  Only two bunk beds can fit into the cell therefore one of the juveniles has to sleep on the floor using mattresses  The toilet is also in the same small room and not separated, which the juveniles have to use in the presence of others  There is a clinic in this section with about five nurses and a doctor who visits four times a week.  According to authorities in the clinic, *“male rape”* is prevalent in the juvenile section with about 2 to 3 reports a week  The juveniles also complained about molestation and assaults especially during routine searches. They complain that authorities insert their fingers into their anus to look for drugs and other forbidden substances.  Only one social worker in the prison.  The Durban Youth Centre has a total of 925 prisoners all males  There is a clinic and a nurse who provides only first aid treatment for minor illnesses and major medical problems are referred to hospital.  Cells are seriously overcrowded including at Stanger prison  and - the inmates sleep virtually in twos on small thin mattresses  Toilet bowls in some of the cells are broken and water was leaking. The prisoners have no beds and have been provided with blankets Only the female section has beds  Presence of social workers, psychologists, religious workers and NGOs.  Structures are very old and very hot in summer  In house clinics in all the prisons visited that provides first aid treatment for minor illnesses.  Clinics lacked laboratory equipment or have very few medicines in the dispensaries (lack of medication).  Provision of basic items like soap, and detergents for washing clothes. | Juveniles in prions were accommodated in a decent environment in some correctional facilities but this was not uniform across all juvenile correctional facilities. Health education awareness programs were being given to the juveniles. .Male rape among the juveniles themselves was reported and a development that is cause for concern as the practice was also likely to continue in the community after release leading to the increase of the prevalence of communicable diseases. The availability of psychological and social services ensured the juveniles welfare and counselling concerns are addressed especially living under such harsh stressful prison conditions. |
|  | African Commission on Human and Peoples’ Rights (ACHPR). Report of the Special Rapporteur on Prisons and Conditions of Detention in Africa. 52nd Ordinary Session, African Commission on Human and Peoples’ Rights. Yamoussoukro, Côte d’Ivoire. 2012.  <http://www.achpr.org/sessions/52nd/>  Accessed 24 Jan 2018 | To assess progress made addressing and implementing recommendations made to different countries taken by the mission and also challenges impacting on implementation | All countries in Africa and including SSA | Presentations, meetings and plenary discussions | As is true for women, most African prison systems besides those in South Africa, Côte d’Ivoire, Mali, Angola and Tunisia lack the resources to house children separately from the adult male population.  Mixing of children with the general prison population can lead to disastrous consequences  First, children imprisoned with the general population must compete with adults for scarce resources such as food.  Second, given that African prisons fail to meet even the most basic minimum standards for adults, hence also fail to meet international standards for juvenile detention.  For example, overcrowding compromises child prisoners’ health and hygiene and exposes them to increased risk of sexual abuse. The educational, developmental, health, and nutritional needs of the juvenile are left unattended. | African Commission on Human and Peoples’ Rights observes that African prisons in general including SSA fail to meet international standards for juvenile detention. Placing these juveniles in situations where they have to compete with adults for virtually everything for survival is unacceptable. Power and control dynamics and the need to survive in such a harsh environment make them choose the latter for survival. This increases their risk vulnerability to abuse. |
| **United States Department of State Bureau of Democracy, Human Rights and Labor 2012 to 2017** | | | | | | |
|  | United States Department of State Bureau of Democracy, Human Rights and Labor. Central African Republic 2012 human rights report. Washington, D.C. 2012  https://www.state.gov/documents/organization/204312.pdf. Accessed 20 Nov 2018. | To report on human rights practices and situation in the country | Central African Republic | Desk review of the reports by Human Rights Unit of the UN Integrated Office in the Central African Republic (BINUCA) and humanitarian NGOs including ICRC | Rudimentary, harsh and life-threatening prison conditions below international standards  Juveniles were sometimes held with adult prisoners  Non-segregation of prisoners with infectious diseases from other inmates  Inadequate basic necessities such as food, clothing,  Lack of basic and emergency medical care  Delays in transferring prisoners along continuum of care unless if critical  Family members supplemented inadequate prison meals  Insufficient potable water.  ICRC and religious groups routinely provided supplies of food, and clothes to prisoners | Common threads running across the countries’ reports included poor physical conditions overcrowding, poor ventilation, hygiene, lack of bedding and prison clothes. Additionally, poor nutrition was also observed including insufficient availability and accessibility of health care. |
|  | United States Department of State Bureau of Democracy, Human Rights and Labor. Mali 2014 human rights report. Washington, D.C. 2014  https://www.state.gov/documents/organization/236592.pdf. Accessed 20 Nov 2018. | To report on human rights practices and situation in the country | Mali | Desk review of independent monitoring reports by HRW such by the Malian Association for Human Rights and ICRC | Overcrowding  Mixing of juvenile prisoners with adults including women  Insufficient prison food in terms of both quality and quantity, and medical facilities were inadequate.  Lack of hygiene and inadequate sanitation posed the most significant threat to prisoners’ health.  Buckets served as toilets.  Not all prisons had access to potable water. |  |
|  | United States Department of State Bureau of Democracy, Human Rights and Labor. Guinea-Bissau 2017 Human Rights Report. Washington, D.C. 2017.  https://www.state.gov/documents/organization/277253.pdf. Accessed 20 Nov 2018. | To report on human rights practices and situation in the country | Guinea-Bissau | Desk review of reports by local and international human rights groups the UN Integrated Peacebuilding Office in Guinea-Bissau and the National Commission for Human Rights visited the prisons in Mansoa and Bafata. | Conditions of confinement described as poor.  No running water, adequate ventilation, lighting, and sanitation.  Juveniles detained held with adult convicted prisoners |  |
|  | United States Department of State Bureau of Democracy, Human Rights and Labor. Comoros 2016 Human Rights Report. Washington, D.C. 2016.  https://www.state.gov/documents/organization/265452.pdf. Accessed 20 Nov 2018. | To report on human rights practices and situation in the country | Comoros | Desk review of International Committee of the Red Cross (ICRC) independent regular monitoring of prisons and a local nongovernmental organization (NGO) focused on prison reform requested to visit | Conditions in prisons described as poor  Juveniles mixed with adult population  Overcrowding and limited access to potable water, inadequate sanitation, insufficient ventilation and lighting, overcrowding  Inadequate medical facilities. |  |
|  | United States Department of State Bureau of Democracy, Human Rights and Labor. Republic of the Congo 2017 Human Rights Report. Washington, D.C. 2017.  https://www.state.gov/documents/organization/277233.pdf. Accessed 20 Nov 2018. | To report on human rights practices and situation in the country | Democratic Republic of Congo | Desk reviews of reports from Human rights NGOs | Old dilapidated infrastructure and prison detention centers conditions harsh and life threatening  Inadequate sanitary conditions, gross overcrowding, and a severe deficit of medical and psychological care.  16- and 17-year-old juvenile prisoners held in the same area as women in Pointe Noire.  In Brazzaville, areas were separate, but sometimes easily accessible with no locked entryways.  In the other 10 prisons, authorities sometimes held juvenile detainees with adult prisoners. |  |
|  | United States Department of State Bureau of Democracy, Human Rights and Labor. Gabon 2017 Human Rights Report. Washington, D.C. 2017.  <https://www.state.gov/documents/organization/277245.pdf>.  Accessed 20 Nov 2018. | To report on human rights practices and situation in the country | Gabon | Desk review of independent monitoring reports by local NGO Malachie that visited prisons. | Harsh and potentially life-threatening prison conditions  Gross overcrowding  Low quality food,  Inadequate sanitation, lack of ventilation  Poor medical care.  Authorities held pretrial detainees with convicted prisoners, juveniles with adults  Juvenile prisoners separated from adults in Libreville and Franceville prisons. |  |
|  | United States Department of State Bureau of Democracy, Human Rights and Labor. Mauritania 2017 Human Rights Report. Washington, D.C. 2017.  https://www.state.gov/documents/organization/277267.pdf. Accessed 20 Nov 2018. | To report on human rights practices and situation in the country | Mauritania | Report by the UN special rapporteur on torture, Mauritanian Human Rights Watch and National Commission on Human Rights (CNDH). Independent monitoring reports through visits by NGOs, diplomats, and international human rights observers. The International Committee of the Red Cross (ICRC) | Harsh life threating prison conditions  Overcrowding, and inadequate sanitary conditions  Food shortages, violence,  inadequate medical care.  Minors in Nouakchott’s Central Prison had contact with adult prisoners, including those convicted of terrorist offenses and other violent crimes  Mixing of prisoners  An Italian NGO operated a detention center for minors which was described as close to meeting international standards |  |
|  | United States Department of State Bureau of Democracy, Human Rights and Labor. Sao Tome and Principe 2017 Human Rights Report. Washington, D.C. 2017.  https://www.state.gov/documents/organization/277281.pdf. Accessed 20 Nov 2018. | To report on human rights practices and situation in the country | Sao Tome and Principe | Desk review of independent monitoring reports by human rights monitors two international entities, domestic charitable groups, including churches | Overcrowding, failing infrastructure and poor ventilation  Inadequate food and sanitation  No juvenile prisoners as of October, but in the past, they have been held together with adults.  Medical care was poor, and the prison lacked basic medicines |  |
|  | United States Department of State Bureau of Democracy, Human Rights and Labor. Senegal 2017 Human Rights Report. Washington, D.C. 2017.  https://www.state.gov/documents/organization/277283.pdf. Accessed 20 Nov 2018. | To report on human rights practices and situation in the country | Senegal | Desk review of reports by independent monitoring visits of local human rights groups, international observers and ICRC | Harsh prison and dentition centre conditions but not potentially life threatening  Poor ventilation and endemic overcrowding, poor sanitation and drainage  Food shortages and poor in both quantity and quality, overcrowding, poor sanitation, and  Juvenile boys were often housed with men or permitted to roam freely with men during the day.  Girls were held together with women.  Inadequate medical care with access controlled by prison guards |  |
|  | United States Department of State Bureau of Democracy, Human Rights and Labor. Sierra Leone 2017 Human Rights Report. Washington, D.C. 2017.  https://www.state.gov/documents/organization/277287.pdf. Accessed 20 Nov 2018. | To report on human rights practices and situation in the country | Sierra Leone | Desk review of assessments by Human rights organizations such as Prison Watch and UN Integrated Peacebuilding Office in Sierra Leone and UN Integrated Peacebuilding the UN Office of the High Commissioner for Human Rights (OHCHR) | Poor infrastructure that lacked of maintenance resulted in prisons not meeting international standards.  Lack of lighting, furniture, beds, sanitary, and medical facilities across the majority of prisons  Inadequate access to food and drinking water poor hygienic conditions  Limited access to medical care  Sometimes mixed juvenile detainees with the adult prison population. |  |
|  | United States Department of State Bureau of Democracy, Human Rights and Labor. Tanzania 2017 Human Rights Report. Washington, D.C. 2017.  https://www.state.gov/documents/organization/277299.pdf. Accessed 20 Nov 2018. | To report on human rights practices and situation in the country | Tanzania | Desk review by the Legal and Human Rights Center (LHRC) and as independent government department, the Commission for Human Rights and Good Governance (CHRAGG) reports of visits to prisons, | Persistent harsh and life -threatening prison conditions  Mixing of Juveniles with adults  Inadequate food, overcrowding, poor sanitation, and insufficient medical care  In several adult prisons, minors were placed in a separate cell but would be mixed with adults during the day and while being transported to court.  While in other prisons children and adults were mixed at all times. |  |
|  | United States Department of State Bureau of Democracy, Human Rights and Labor.  Togo human rights report 2017. Washington, D.C. 2017.  https://www.state.gov/documents/organization/277301.pdf. Accessed 24 Nov 2018. | To report on human rights practices and situation in the country | Togo | Monitoring reports on visits by local nongovernmental organizations accredited by the Ministry of Justice | There were 45 juveniles held in the Brigade for Minors facility. Medical facilities, unhealthy food, poor sanitation, ventilation, and lighting were inadequate  Prisoners did not have access to potable water, and disease was reported as widespread. |  |
|  | United States Department of State Bureau of Democracy, Human Rights and Labor. Madagascar 2017 Human Rights Report. Washington, D.C. 2017.  https://www.state.gov/documents/organization/277261.pdf. Accessed 19 Nov 2018. | To report on human rights practices and situation in the country | Madagascar | Desk review of independent monitoring reports through visits by NGOs, diplomats, and international human rights observers and the International Committee of the Red Cross (ICRC) | Deteriorating prison infrastructure that often-lacked sanitation facilities and potable water  Disease as a result of poor hygiene resulted and infestations of insects and rodents.  Conditions in prisons described as poor with overcrowding  Juveniles mixed with adult population during the day with some sharing dormitories with adults  Limited access to potable water, inadequate sanitation, insufficient ventilation and lighting, overcrowding  Inadequate medical facilities. |  |
|  | Cote D’ivoire 2017 Human Rights Report.  https://www.state.gov/documents/organization/277235.pdf. Accessed 20 Nov 2018. | To report on human rights practices and situation in the country | Cote D’ivoire | Desk review of independent monitoring reports through visits by United Nations and local and international NGOs | Prison conditions poor and life-threatening  Overcrowding with inadequate sanitary conditions, worsened by lack of portable water  Lack of medical care.  Philanthropic organizations sometimes financed prisoners’ medical care.  Juveniles were held with adults in the same cells in some prisons, pretrial detainees were accommodated together with convicted prisoners.  . |  |
|  | United States Department of State Bureau of Democracy, Human Rights and Labor. Equatorial Guinea human rights report 2017.Washington, D.C. 2017.  https://www.state.gov/documents/organization/277239.pdf. Accessed 24 Nov 2018. | To report on human rights practices and situation in the country | Equatorial Guinea | Reports of visits by NGOs UNICEF visits to youth rehabilitation centers in Centro Sur and Riaba | Overcrowded, dirty prison cells with no mattresses  Ventilation and lighting not always adequate  Prevalence of malaria, typhoid, tuberculosis, hepatitis C, HIV/AIDS  Minors had separate sleeping quarters and bathrooms but shared a common area for meals with adults.  Inconsistent provision of medical care to prisoners and detainees’ Inadequate basic meals and inadequate food poor in both quantity and quality  . |  |
|  | United States Department of State Bureau of Democracy, Human Rights and Labor. Rwanda 2017 Human Rights Report. Washington, D.C. 2017.  https://www.state.gov/documents/organization/277279.pdf. Accessed 20 Nov 2018. | To report on human rights practices and situation in the country | Rwanda | Desk review of Human Rights Reports | Prison conditions were reported as ranging from harsh to life threatening and failing to meet international standards  Officials holding children together with adults in Muhanga, Mudende, and Gikondo.  In other prisons juvenile prisoners were sometimes held in separate facilities, such as in Mbazi that had marginally better conditions than the facilities for adults.  Juveniles only facility in Nyagatare came close to meeting international norms. |  |
|  | United States Department of State Bureau of Democracy, Human Rights and Labor. Swaziland Human Rights Report 2017. Washington, D.C. 2017.  https://www.state.gov/documents/organization/277297.pdf. Accessed 24 Nov 2018. | To report on human rights practices and situation in the country | Swaziland | Investigative journalism reports by AllAfrica Global Media and Swazi Observer. Reports by international NGO Save the children and local NGOs | Harsh prison conditions due to overcrowding,  Food shortages, physical abuse, and inadequate sanitary conditions reported  inadequate and medical care.  Reports of juvenile prisoners inhuman and degrading treatment at the juvenile centers, including physical assault and strip searches of female juvenile prisoners. |  |
|  | United States Department of State Bureau of Democracy, Human Rights and Labor. Seychelles 2017 Human Rights Report. Washington, D.C. 2017.  https://www.state.gov/documents/organization/277285.pdf. Accessed 20 Nov 2018. | To report on human rights practices and situation in the country | Seychelles | Desk review of human rights independent reports by international and local NGOs based observation visits during monitoring of prison conditions and the UN Office on Drugs and Crime (UNODC) | Juvenile pretrial detainees and convicted prisoners were held together with adult prisoners  Prison conditions and overcrowding in Montagne Posee Prison, the main prison out of the three institutions |  |
|  | United States Department of State Bureau of Democracy, Human Rights and Labor. Cabo Verde 2017 Human Rights Report. Washington, D.C. 2017. https://www.state.gov/documents/organization/277221.pdf. Accessed 20 Nov 2018. | To report on human rights practices and situation in the country | Cape Verde | Desk review of reports of formal visits records by international human rights monitors to the prisons and individual prisoners, local NGOs and members of the press | Harsh and potentially life-threatening physical conditions due to gross overcrowding and inadequate housing and sanitation.  The Orlando Pantera Center housed juvenile detainees who were under age 16 at the time of sentencing  In the Fogo regional prison, all 11 cells and the isolation cells housed youth and adults together  Inadequate bedding with some prisoners having mattresses and beds while some slept on thin blankets on concrete floors. Shower and toilet facilities were inadequate and unsanitary  Distribution personal hygiene kits and prioritization of improvements to the showers and toilets facilities |  |
|  | United States Department of State Bureau of Democracy, Human Rights and Labor. Botswana human rights report. Washington, 20`17.  https://www.state.gov/documents/organization/277215.pdf. Accessed 24 Nov 2018. | To report on human rights practices and situation in the country | Botswana | Desk review of human rights independent reports by international and local NGOs based observation visits to prisons. The International Committee of the Red Cross visited prisons. | Prison and detention center conditions reported as generally meeting international standards and continued to improve with further reduction of inmate overcrowding.  Authorities occasionally held juveniles with adults, although only for a few days while awaiting transport. | Conditions of incarceration close to meeting international standards due to reduction in number of inmates that in turn has decreased overcrowding |
